# Impact of Heat Stress on Oocyte Developmental Competence and Pre-Implantation Embryo Viability in Cattle

**DOI:** 10.3390/ani14152280

**Published:** 2024-08-05

**Authors:** Javier A. Gómez-Guzmán, Gaspar M. Parra-Bracamonte, Miguel A. Velazquez

**Affiliations:** 1 Centro de Biotecnología Genómica, Instituto Politécnico Nacional, Reynosa 88710, Tamaulipas, Mexico; jgomezg1800@alumno.ipn.mx (J.A.G.-G.); gparra@ipn.mx (G.M.P.-B.); 2 School of Natural and Environmental Sciences, Newcastle University, Newcastle upon Tyne NE1 7RU, UK

**Keywords:** heat stress, cattle, oocyte competence, ovarian follicle, pre-implantation period, embryo viability, uterus, oviducts

## Abstract

**Simple Summary:**

Heat stress is a major problem for the health and productivity of livestock, including cattle. Since the 1950s, researchers have studied how high environmental temperature affects cattle fertility through both laboratory and field studies. When a cow’s rectal or vaginal temperature goes above 39.1 °C, it is considered heat-stressed, which can harm its ability to reproduce. However, there is not enough information about temperatures in the uterus, oviducts, and ovarian follicles, especially in lactating dairy cows, to ensure current laboratory models are accurate. These data are crucial for understanding how heat stress impacts oocyte (egg) development and early embryo survival in cattle. Additionally, it is important to improve live animal stress models to correctly identify when cows are truly experiencing heat stress, shown by signs like increased panting, body temperature, and heart rate, rather than just assuming they are stressed based on exposure to a high temperature–humidity index. Improving these models will make them more reliable and help identify cattle that can better tolerate heat.

**Abstract:**

Rectal and vaginal temperatures are utilised in both in vivo and in vitro models to study the effects of heat stress on oocyte competence and embryo viability in cattle. However, uterine temperature increases by only 0.5 °C in heat-stressed cows, significantly lower than simulated increases in in vitro models. Temperature variations within oviducts and ovarian follicles during heat stress are poorly understood or unavailable, and evidence is lacking that oocytes and pre-implantation embryos experience mild (40 °C) or severe (41 °C) heat stress inside the ovarian follicle and the oviduct and uterus, respectively. Gathering detailed temperature data from the reproductive tract and follicles is crucial to accurately assess oocyte competence and embryo viability under realistic heat stress conditions. Potential harm from heat stress on oocytes and embryos may result from reduced nutrient availability (e.g., diminished blood flow to the reproductive tract) or other unidentified mechanisms affecting tissue function rather than direct thermal effects. Refining in vivo stress models in cattle is essential to accurately identify animals truly experiencing heat stress, rather than assuming heat stress exposure as done in most studies. This will improve model reliability and aid in the selection of heat-tolerant animals.

## 1. Introduction

It is projected that climate change will increase the exposure to episodes of heat stress worldwide [1,2,3,4], affecting the performance, health, and welfare of domestic livestock species, including cattle [4,5,6]. Heat stress arises when a combination of climate variables—such as low wind velocity and high air temperature, relative humidity, and solar radiation —creates a microenvironment that disturbs the thermal equilibrium of the body, moving the internal body temperature out of the thermoneutral zone into the upper critical temperature zone [7]. The upper critical temperature is the ambient temperature above which heat evaporative mechanisms need to be activated to maintain a stable internal body temperature compatible with optimal physiological function [8]. Previous studies conducted on dairy cattle in the 1980s and 1990s in northern Israel [9] and Arizona, USA [10], reported upper critical air temperatures of 25.0–26.0 °C and 24.0–27.0 °C, respectively. More recently, an upper critical air temperature of 28.4 °C has been suggested based on data from Florida, USA, and the differences between studies were attributed to differences in husbandry (e.g., the use of mitigation strategies for heat stress) and partial adaptation to heat stress of the analysed herd [11]. However, these upper critical air temperatures were calculated in subtropical areas, and may differ for dairy cattle raised in temperate climates, particularly those exposed to heatwaves during summers in temperate regions such as the UK [12,13,14], where temperatures of 40 °C were recorded for the first time in July 2022 [15]. Furthermore, genetic selection for productive traits in the cattle industry can result in a diminished genetic ability to adapt to local environmental changes [16] and could potentially exacerbate the vulnerability of dairy cattle to heat stress associated with increased metabolic heat generation from high milk production [17]. Indeed, a marked increase in respiration rate can be observed at 21 °C in cattle [18], and a study in Sweden revealed a sharp decline in milk production in dairy cattle at environmental temperatures of 22.0–23.0 °C [19].

The consequences of heat stress extend beyond reduced milk production, affecting animal welfare [20,21] and, in extreme cases, leading to the death of animals [22,23,24,25,26,27]. Heat stress also has an economic impact on cattle production. St-Pierre et al. [28] reported over a decade ago annual losses in the USA of approximately USD 897 million for the dairy industry and USD 369 million for the beef industry. More recently, Hristov et al. [29] suggested that milk production losses in dairy herds from the Northeast in the USA are expected to be 0.40% in 2050 and 1.02% by the end of the century, representing an economic loss of USD 49.1 and USD 125.8 million per year, respectively. In another study in the USA, it was predicted an annual national decline in milk production of 1.4 and 1.9 kg/day in 2050 and 2080 with economic losses of USD 1.7 billion and USD 2.2 billion, respectively [30]. Similarly, research in Germany and Spain estimated a 2.8% reduction in milk yield due to heat stress, resulting in financial losses of about 5.4% of the monthly income, especially during the summer season [31]. Latest global prediction models indicate that heat stress will also lead to significant financial losses associated with decreased milk and beef production in Sub-Saharan Africa during the middle and end of the century. A similar scenario is expected in Central America [5]. While these estimates typically rely on the negative impact of heat stress on feed intake, which in turn decreases milk production, it is well documented that in cattle, heat stress can be detrimental to oestrus cyclicity [32,33], oocyte quality [33,34], and pre-implantation embryo development [35,36,37,38,39], thus negatively affecting their reproductive performance.

A key component for successful reproduction is the ability of the oocyte to maintain optimal development and maturation to achieve fertilisation and subsequent early embryo development. Another critical aspect for reproductive success is the provision of a microenvironment in the oviduct and uterus compatible with sound pre-implantation embryo development. Indeed, the periconceptional period is a critical developmental period [40,41] during which heat stress can exert short- and long-term detrimental effects on the resultant embryos and offspring [42]. In this review, we provide an extensive discussion of the current understanding of the cellular and molecular alterations exerted by heat stress on oocyte developmental competence and embryo viability in cattle. Additionally, we will critically evaluate the reliability of in vitro models in this context.

## 2. Impact of Heat Stress before Resumption of Meiosis in Cattle

While oogonial stem cells (OSCs) have been isolated from adult mice ovaries and are capable of differentiating into fertilisable oocytes and producing offspring via intraovarian transplantation of OSCs [43,44], the role of this putative postnatal oogenesis in maintaining a pool of primordial follicles in vivo remains uncertain, particularly considering that OSCs represent only 0.014% of the cell population per ovary in mice [44]. Moreover, there is controversy regarding the presence of OSCs in the ovaries of humans and other species [45,46]. Therefore, the current evidence does not sufficiently challenge the mainstream notion that postnatal ovaries in mammalian species, including cattle, possess a fixed, non-renewable pool of primordial follicles [47,48]. Primordial follicles are formed during foetal development [49], and each primordial follicle contains a primordial oocyte surrounded by pre-granulosa cells (i.e., a single layer of flattened epithelial cells) halted at the diplotene stage of the first meiotic division [50,51]. Upon activation, the primordial follicle undergoes several developmental stages starting with the transition into a primary follicle, followed by progression to a secondary follicle, with subsequent transformation into an antral follicle [52].

The effects of in vivo heat stress exposure on germinal vesicle (GV)-stage oocytes in cattle have been studied using oocytes collected by ovum pick-up (OPU) from live animals, coupled with in vitro embryo production (IVEP). During OPU, immature oocytes (i.e., in the GV stage) are collected and subsequently subjected to in vitro maturation (IVM), in vitro fertilisation (IVF), and in vitro embryo culture (IVEC) to produce blastocysts. Alternatively, immature oocytes can also be obtained from ovaries collected at the local abattoir [53]. With this model, it was reported that compared to the cool season, abattoir-derived oocytes from Holstein cows produce fewer blastocysts during the hot season in the USA [54,55], Israel [56,57,58,59], and Portugal [60,61]. In South Korea [62], Japan [63], and Uruguay [64], GV-stage oocytes from beef cattle collected at the abattoir during the summer showed a reduced ability to form blastocysts following IVM. Using abattoir-derived Holstein GV-stage oocytes, it was also demonstrated that embryo development following oocyte chemical activation was impaired during the hot season [65]. This seasonal temperature effect on IVEP was not detected with abattoir-sourced oocytes from beef cattle by researchers in Brazil, but the same group reported lower blastocyst production during the hot season with OPU-derived oocytes from Holstein heifers and cows [66]. Similarly, a retrospective study in Brazil with Holstein cows found that exposure to temperatures above 15 °C on day 30 prior to OPU yielded fewer viable oocytes and produced fewer embryos compared to cows kept below 15 °C [67]. However, no effect on blastocyst production was reported when heat stress took place on day 60 or 90 before OPU, or on the day of OPU [67]. Studies conducted in Mexico involving Holstein cattle found that embryo production via OPU-IVEP was lower during the warmest months of the year, with a more pronounced effect observed in cows compared to heifers [68,69].

### 2.1. Cellular and Molecular Effects of Heat Stress on Bovine Germinal Vesicle-Stage Oocytes

Previous research with seasonal studies reported that transcript expression of GV-stage oocytes is not affected, but the gene expression of oocytes following IVM and that of resultant embryos is impacted. As such, in vitro matured oocytes derived from GV-stage oocytes sourced during the hot season showed decreased expression of genes associated with modulation of mitochondrial electron transport chain (*ND2*, *SDHD*, *CYTB*, *COXII*, *ATP5B*) [58], regulation of oocyte maturation (*C-MOS*, *GDF9*), and control of embryonic cell lineage differentiation (*POU5F1*) [56,57]. The downregulation of *POU5F1* was further observed at the two-cell [70], four-cell, eight-cell, and blastocyst stages [57]. Similarly, GV-stage oocytes collected during warm months produced embryos with lower expression of genes involved in the regulation of cell membrane permeability (*CX43*), DNA methylation (*DNMT1*), and cellular stress response (*HSPA14*) [61]. However, other studies have shown that transcript activity in GV-stage oocytes varies significantly between hot and cool seasons. For instance, immature oocytes harvested from dairy cows and heifers in the summer displayed diminished gene expression linked to mitochondrial activity (*NRF1*, *POLG*, *POLG2*, *PPARGC1A*, *TFAM*), programmed cell death (*BAX* and *ITM2B*), and oocyte maturation (*FGF16* and *GDF9*) [71]. A recent RNA sequencing analysis of GV-stage oocytes from Angus cows collected in the summer identified changes in the expression of genes involved in steroid biosynthetic processes, oxidation-reduction, and mitophagy in response to mitochondrial depolarisation, linking them to pathways associated with glucocorticoid biosynthesis, apoptosis signalling, and the HIPPO signalling pathway, which regulates cell proliferation, differentiation, and survival. Following IVM, the resultant MII oocytes exhibited changes in the transcriptional regulation of genes involved in the MAPK signalling cascade, melanosome organisation, and negative regulation of transcription. This altered gene expression influenced pathways related to Oct4-mediated pluripotency signalling, Wnt/beta-catenin signalling, and melatonin degradation [72].

Other variables of oocyte quality, such as mitochondrial distribution, can be impaired in in vitro matured oocytes originating from summer-collected GV-stage oocytes without altering their mitochondrial DNA (mtDNA) copy number [58]. Likewise, the number of mtDNA copies in GV-stage oocytes was not affected by season in reproductively healthy dairy heifers and cows [71]. In beef cows, immunostaining analyses indicated no seasonal effect on global DNA methylation (5-methylcytosine) and DNA hydroxymethylation (5-hydroxymethylcytosine) in GV-stage oocytes [72], nor the proportion of MII oocytes following IVM [64,72]. Similarly, the ability of GV-stage oocytes to achieve fertilisation following IVM-IVF does not appear to be affected by seasonally high temperatures [64]. Nevertheless, an increase in apoptosis of immature cumulus–oocyte complexes (COCs) from beef cattle was observed in the summer months, which persisted following IVM [64].

At the embryo level, blastocysts derived from GV-stage oocytes obtained during warm months did not exhibit changes in apoptosis level [64,71] or cell number [54,64] in dairy and beef cattle. A recent study used time-lapse analysis to compare the developmental potential of GV-stage oocytes between the hot and cold seasons. It was found that although the second embryonic cleavage was delayed, the overall timeline to reach the blastocyst stage was not influenced by seasonal temperatures. However, the occurrence of abnormal cleavage was higher during the hot season and was characterised by zygotes dividing into two blastomeres of different sizes. Consequently, a greater percentage of blastocysts originated from normally cleaved embryos during the cold season than during the hot season. Gene expression analysis of the resultant embryos indicated that the transcript abundance of genes involved in immune response regulation (*STAT1*) and cellular stress (*HSF1*) increased, while the expression of *ZP3* (sperm binding receptor) decreased. [59]. Although it might be argued that these studies could be detecting an effect of season that is not necessarily due to heat stress (e.g., nutrient availability), there is good in vivo experimental evidence demonstrating the detrimental effects of high temperatures on GV-stage oocyte competence. For instance, using environmental chambers, it was reported that Gir cows exposed to temperatures of 30–38 °C for 28 days before OPU displayed a decrease in weekly blastocyst production during the following 105 days after heat stress exposure [73].

### 2.2. Contribution of In Vitro Models to Assess the Impact of Heat Stress on Germinal-Vesicle-Stage Oocytes

In vitro models have also illustrated the detrimental effects of heat stress on immature oocytes, where culturing GV-stage oocytes under 41.0 °C with meiosis inhibitors to prevent the spontaneous resumption of meiosis resulted in decreased embryo production following IVM, IVF, and IVEC [56,74,75]. But this detrimental effect was not associated with the ability of heat-stressed GV-stage oocytes to reach metaphase II or achieve fertilisation following IVM [74]. Furthermore, the resultant matured oocytes also showed increased apoptosis and decreased mitochondrial activity, but the resultant embryos did not show alterations in cell number or level of apoptosis [75]. In another study, exposing oocytes from early antral follicles with diameters of 0.5–1 mm to temperatures ranging from 39.5 to 40.5 °C for 9 hours (h) daily over 12 days did not affect their ability to achieve in vitro maturation, but completely impaired their capacity to reach the blastocyst stage [76]. In vitro models have also shown that preantral development can be impaired by heat stress. Using Calcein AM, a membrane-penetrating dye for labelling live cells, in combination with ethidium homodimer-1, which penetrates only dead cells, it was reported that preantral follicles (i.e., follicles enclosed in ovarian tissue) displayed lower viability when exposed to 41 °C for 12 h a day for 7 days, along with increased production of reactive oxygen species (ROS) [77]. Similarly, the viability of preantral secondary follicles, as shown by trypan blue intake (a dye that penetrates only dead cells), decreased when they were exposed to 41 °C for 8 h daily over a period of 7 days. This was accompanied by a reduction in follicle diameter and increased expression of genes associated with apoptosis (*BAX*) and protection against oxidative stress (*SOD1*) [78]. More recently, exposing ovarian cortex fragments to 41 °C for 2 h decreased the number of primordial follicles, while the counts of primary and secondary follicles remained unchanged. Heat-stressed primordial follicles also exhibited reduced follicle diameter and fewer granulosa cells. Additionally, increased levels of apoptosis were observed, specifically in primordial and primary follicles [79]. While no in vivo bovine model has yet analysed the effect of heat stress on the viability of primordial follicles, in rabbits, in vivo exposure to thermal stress (31 °C) did not decrease the number of primordial follicles, but it did increase the incidence of vacuolisation in these follicles [80]. Contrary to earlier assumptions of their heat resistance [34,81], these findings suggest that primordial follicles might be more susceptible to heat stress than previously believed.

Granulosa cells from small follicles have also been analysed, with fluorescence assays revealing increased production of ROS and apoptosis in heat-stressed granulosa cells exposed to 40 °C or 41 °C for 2 h in vitro [82,83]. Moreover, RNA-sequencing of these granulosa cells revealed that heat stress altered pathways involving heat shock proteins, apoptosis, and oxidative stress [82]. The same team also explored the transcriptome and metabolome of granulosa cells from small follicles using a model of heat exposure at 43 °C for 2 h in vitro. Dysregulation of gene expression and metabolite production was observed in this model [84,85,86], but the practical relevance of this information for bovine reproduction is uncertain as it is extremely unlikely that a cow or heifer will reach a rectal temperature of 43 °C under natural exposure to heat stress. Although some dairy cows can apparently reach rectal temperatures exceeding 42 °C when purposely exposed to unshaded conditions during the hot season in Florida, USA [87], such high rectal temperatures are typically observed in pathological cases, such as cattle with syndactyly. During one week of exposure to 37 °C in a climate chamber, cows and heifers with this condition could reach rectal temperatures of 44.5–45 °C before succumbing, whereas control animals maintained a rectal temperature of around 40 °C [88]. Furthermore, IVM at 43 °C for 12 h completely abolished the ability of oocytes to reach the blastocyst stage [89].

## 3. Impact of Heat Stress during Oocyte Meiotic Maturation in Cattle

Oocyte maturation encompasses a series of molecular and cellular events that provides the oocyte with the ability to achieve fertilisation and form a competent embryo [90,91,92]. During maturation, GV breakdown takes place, with the subsequent completion of the first meiotic division resulting in the formation of the first polar body, followed by an arrest at the metaphase II stage of the second meiotic division. At this point, nuclear and cytoplasmic maturation have been achieved, and the oocyte is ovulated [92,93,94].

Research conducted in environmental chambers with sheep in the 1960s suggested that oocyte maturation is a key developmental period affected by heat stress, as ewes exposed to high temperatures at the time of breeding (~24 h before fertilisation) experienced a significant increase in oocyte degeneration and embryo loss [95,96]. In cattle, it was found that exposing superovulated heifers to 42 °C for 10 h in an environmental chamber during oestrus, but before artificial insemination, was sufficient to decrease the proportion of embryos with good morphological quality and their cell number [97]. Similarly, a decrease in pregnancy rate was observed when dairy cows were exposed to a temperature–humidity index (THI) of more than 80 on the day before artificial insemination [98]. Heat stress can induce changes in the reproductive physiology of cattle, including dysregulated luteinising hormone (LH) and follicle-stimulating hormone secretion [99,100], diminished follicular [101,102] and luteal [102,103] blood flow, decreased dominant follicle size [102,104,105,106,107], atypical follicular development dynamics [73,108,109,110], impaired ovarian steroid production [111,112,113], unbalanced intrafollicular protein content [107,114], and delayed regression of the corpus luteum [105,108]. Despite contradictory evidence for some of these physiological variables, such as the lack of effect of heat stress on steroid production [115] and luteal function [116,117], all these ovarian regulatory factors can ultimately impair follicular development and oocyte quality, making it challenging to determine whether there is a direct effect of high temperature on oocyte maturation.

In this context, IVEP cycles have proved useful for analysing in detail the effects of heat stress exposure during oocyte maturation. The majority of in vitro models of heat stress have confirmed a decrease in blastocyst formation when oocytes are exposed to maturation temperatures ranging from 39.5 to 43 °C, with 40 to 41.5 °C being the most commonly used range. However, impaired blastocyst production is not always associated with a decrease in embryo cleavage (Table 1). Furthermore, outcomes of in vitro models have been contradictory in some cases. For example, Alves et al. [118] reported no blastocyst production when IVM took place at 40 °C for 24 h, whereas Cebrian-Serrano et al. [119] found an improvement in blastocyst formation with IVM at 41 °C for 20 h. Even within the same research group, contrasting results have been reported, where both no effect and impaired blastocyst production were observed when oocytes were in vitro matured during the first 12 h of IVM at 41 °C [120,121].

Most studies examining the effect of heat stress on oocyte fertilising ability have reported a reduction in fertilisation rates (Table 1), suggesting that at least 12 h of exposure to temperatures between 40 and 41 °C during IVM is required to significantly impair fertilisation [122]. At the cell kinetics level, in vitro research has shown that COCs exposed to 41 °C for 12 h exhibited a lower ability to achieve compaction [123]. Similarly, time-lapse studies have revealed that oocytes matured for 22 h at 41.5 °C displayed a delay in first and second cleavage, resulting in an increased timing for blastocyst formation [124]. Interestingly, applying thermal stress to compact morulae derived from heat-stressed oocytes decreased their ability to reach the blastocyst stage [123]. This finding might be pertinent to consider when using in vivo-produced morulae for embryo transfers as a strategy to mitigate the effects of heat stress [125]. However, the detrimental effect on embryo viability from this second hit of heat stress seems to be absent when the standard IVEP is less than 20%. Furthermore, in these IVEP cycles, exposing morulae derived from non-heat-stressed oocytes to thermal stress (i.e., 41 °C for 12 h) decreases their potential to progress to the blastocyst stage [123], a situation not observed in vivo [87] or in IVEP cycles with good embryo production (i.e., >20%) [120,126,127,128]. The precise contributing factors for this low embryo production are unknown, but it has been attributed to an initial random low developmental competence of the GV-stage oocytes present in abattoir material, and to technical factors (e.g., batch variation in the culture medium used) present in some IVEP replicates [123].

### 3.1. Cellular and Molecular Effects of Heat Stress on Bovine Oocyte Competence during In Vitro Maturation

Impaired blastocyst formation during IVM under heat stress is associated with compromised oocyte maturation. As such, the percentage of oocytes displaying a polar body was decreased at temperatures ranging from 41 to 41.5 °C [119,129], while other studies reported no effect on polar body extrusion within a 40–41 °C range [130,131,132]. Further exposure to 42–43 °C also affected the formation of the first polar body during IVM [131]. However, the detection of a polar body following IVM may not be an accurate way of determining oocyte maturation status, as the decreased presence of a polar body in heat-stressed oocytes was not linked to the proportion of metaphase II (MII) oocytes determined by nuclear staining [129]. Indeed, it has been observed in humans that oocytes with a visible polar body may not necessarily be mature [133].

**Table 1:** In vitro bovine models analysing the effect of heat stress during oocyte maturation on blastocyst production.

Temperature (°C)/CO_2_◆	Exposure Times (h)	Oocyte Maturation Rate		Fertilisation Rate		Cleavage Rate		Blastocyst Rate		Blastocyst Quality	Reference
•C: 39🛇 HS: 41, 42	12, 24		NR		NR	NR	C: 81% HS: 15–74%	↓ ↔ ^a^	C: 29% HS: 1–10%	NR	[121]
•C: 39🛇 HS: 41	12		NR		NR	NR	C: 80% * HS: 70% *	↓↔ ^b^	C: 35–46% * HS: 18–41% *	NR	[120]
•C: 38.5🛇 HS: 41	12		NR		NR	↔	C: 75% * HS: 72% *	↓	C: 24–28% * HS: 14–20% *	↓Cell number	[134]
•C: 38.5🛇 HS: 40, 41	12		NR		NR	↓	C: 75–80% * HS: 60–65% *	↓	C: 17–27% * HS: 9–16% *	↑Cell number ↔Apoptosis	[135]
•C: 38.5🛇 HS: 41, 43	12		NR		NR	↓	C: 80–83% * H: 12–61% *	↓	C: 24–27% * H: 0–14% *	↔Cell number ↔Apoptosis	[89]
•C: 38.5🛇 HS: 41	6, 12, 24	↔ ^1,2^	C: 83% HS: 83–89%		NR	↔	C: 66% HS: 61%	↓ ^c^	C: 31% HS: 17%	↔Cell number	[129]
•C: 38.5↺ HS: 40, 41	12	↓ ^2^	C: 56% H: 19%	↓	C: 72% H: 33%		NR		NR	NR	[136]
•C: 38.5⊘ HS: 41	12		NR		NR	↔	C: 71% HS: 70%	↓	C: 29% * HS: 14% *	NR	[137]
•C: 39⊘ H: 41	21		NR		NR	↓	C: 85% * H: 75% *	↓	C: 33% * H: 16% *	↔Cell number ↑Apoptosis	[138]
•C: 38.5⊘ H: 41	12	↔ ^2^	C: 70% * H: 73% *		NR	↔	C: 84% H: 79%	↓	C: 20% H: 14%	↓Cell number	[139]
•C: 38.5⊘ H: 41	22		C: 92% * H: 74% *		NR	↓	C: 92% * H: 84% *	↓	C: 25% * H: 14% *	↔Cell number ↔Apoptosis	[140]
•C: 38.5⊘ HS: 41	12		NR		NR	↔	C: 80% HS: 80%	↓	C: 29% HS: 19%	NR	[141]
•C: 38.5⊘ HS: 41	12		NR	↔	C: 75% H: 77%	↔	C: 76% HS: 64%	↓	C: 35% HS: 18 *–22%	NR	[142]
•C: 38.5⊘ H: 39.5–40.5	⮥20	↓ ^2^	C: 72% H: 34%		NR	↔	C: 89% H: 81%	↓	C: 31% H: 18%	NR	[143]
•C: 38.5⊘ HS: 40	24		NR		NR	↓	C: 68% HS: 31%	↓	C: 43% HS: 0%	NR	[118]
•C: 38.5⊘ HS: 41	17		NR		NR	↔	C: 75% HS: 72%	↓	C: 37% HS: 23%	↓Cell number ↑Apoptosis	[144]
•C: 38.5⊘ HS: 41, 41.5	20, 24	↓ ^1^	C: 81–83% HS: 66–54%		NR	↓	C: 78% HS: 60%	↓↑ ^d^	C: 17–23% HS: 10–23%	↓↔Cell number	[119]
•C: 38.5⊘ HS: 41.5	22	↓ ^2^	C: 59% HS: 12%		NR	↓	C: 75% * HS: 57% *		NR	NR	[145]
•C: 38.5⊘ HS: 39.5,40.5	24	↓ ^2^	C: 78% HS: 21–8%		NR	↓	C: 70% H: 35–15%	↓	C: 48% HS: 20–9%	Altered gene expression	[61]
•C: 38.5⊘ HS: 41	14	↓ ^2^	C: 78% * H: 54% *		NR	↔	C: 68 *–83% H: 59 *–62%	↓	C: 20–33 *% H: 7–16 *%	↓Cell number ↑Apoptosis	[146]
•C: 38.5⊘ HS: 41	12	↔ ^2^	C: 75% * HS: 70% *		NR	↔	C: 73% H: 70%	↓	C: 47% H: 31%	↓Cell number ↑Apoptosis	[147]
•C: 38.5⊘ HS: 40.5	1, 4, 12	↔ ^2^	C: 83% H: 63–84%		NR	↔	C: 64% H: 70–73%	↓↔ ^e^	C: 31% H: 21–29%	↑Apoptosis	[148]
•C: 39⊘ HS: 41.5	1		NR		NR	↔	C: 67% H: 82%	↔	C: 21% H: 15%	NR	[149]
•C: 38.5⊘ HS: 41	14		NR		NR	↓	C: 76% H: 52%	↓	C: 26% H: 17%	NR	[150]
•C: 38.5⊘ HS: 41	12		NR		NR	↔	C: 70% H: 69%	↓	C: 29% H: 19%	↔ATP content	[151]
•C: 38.5⊘ HS: 41	6, 12, 18, 22	↓ ^2^	C: 75% * HS: 35–60% *	↓↔ ^f^	C: 53% * HS: 19–45% *	↓↔ ^f^	C: 72% * HS: 62–30% *	↓↔ ^f^	C: 30% * HS: 9–25% *	↓Cell number	[122]
•C: 38.5↺ H: 41.5	12		NR		NR	↔	C: 74% H: 64%	↓	C: 34% H: 21%	↔Cell number ↑Apoptosis	[152]
•C: 38.5⊘ HS: 41	12		NR	↓	C: 84% H: 74%	↓	C: 67% * H: 53% *	↓	C: 20% * H: 10% *	↔Cell number ↔Apoptosis	[153]
•C: 38.5↺ HS: 41	14		NR		NR	↓	C: 65% * HS: 52% *	↓	C: 26% HS: 22%	NR	[154]
•C: 38.5⊘ H: 41	14		NR		NR	↓	C: 71–78% * H: 59–66% *	↓	C: 22 *–30% H: 13 *–14%	Unaltered gene expression	[155]
•C: 38.5⊘ H: 41	12	↓ ^2^	C: 81% H: 65%		NR	↓	C: 78% H: 61%	↓	C: 39% H: 16%	Altered gene expression	[156]
•C: 38.5⊘ H: 40.5	⮥6		NR		NR	↓	C: 89% * H: 60% *	↓	C: 31% * H: 26% *	↔Cell number ↓IFNT, ↑ROS	[157]
•C: 38.5↺ H: 41	16		NR		NR	↔	C: 70% * H: 62% *	↓	C: 24% * H: 17% *	NR	[158]
•C: 38.5⊘ H: 40	24	↔ ^1^	C: 80% H: 74%		NR	↓	C: 76% H: 26%	↓	C: 32% H: 11%	NR	[130]
•C: 39⊘ H: 41	6		NR		NR	↓	C: 81% H: 73%	↓	C: 33% H: 18%	Altered gene expression	[159]
•C: 38.5⊘ H: 41.5	22	↓ ^2^	C: 64% * H: 14% *		NR	↓	C: 80% * H: 58% *	↓	C: 21% * H: 5% *	Unaltered gene expression	[124]
•C: 38.5⊘ H: 41	12		NR		NR	↓	C: 79% H: 61%	↓	C: 31% H: 13%	Altered gene expression	[160]
•C: 38.5⊘ H: 41	12	↔ ^2^	C: 70% H: 66%		NR	↔	C: 70 H: 67	↓	C: 39% H: 16%	↓Cell number	[161]
•C: 38.5⊘ H: 41.5	4, 6		NR		NR	↔	C: 68% H: 75–75%	↔	C: 23% H: 27–29%	NR	[162]
•C: 39⊘ H: 41	6		NR		NR	↓	C: 82–85% H: 70–76%	↓	C: 30–35% H: 16–24%	Altered gene expression	[163]
•C: 39⊘ H: 41	6		NR		NR	↓	C: 84% H: 78%	↓	C: 33% H: 24%	Altered gene expression	[164]
•C: 39⊘ H: 41	24	↔ ^1^	C: 57% H: 60%		NR	↔	C: 65% H: 62%	↔	C: 11% H: 11%	↓Cell number ↑Apoptosis	[131]
•C: 38.5⊘ HS: 41	12	↓ ^1^	C: 84% HS: 61%		NR	↓	C: 80% HS: 58%	↓	C: 42% HS: 23%	Altered gene expression	[165]
•C: 38.5⊘ HS: 39.5, 40.5	23	↓ ^2^	C: 61% H: 23–40%		NR	↓↔ ^g^	C: 84% H: 58–66%	↓↔ ^g^	C: 22% H: 8–24%	↔Cell number	[166]

C = control, HS = heat stress, NR = not reported, ↓ = significant decrease compared to control, ↑ = significant increase compared to control, ↔ = no difference between groups, IFNT = interferon tau, ROS = reactive oxygen species. CO_2_◆ indicates whether the study maintained 5% CO_2_ (⊘) or increased it (↺) during IVM heat stress conditions. ^1^ Maturation rate analysed via extrusion of the first polar body. ^2^ Maturation rate analysed via nuclei staining. * Percentages are approximate, extracted from bar charts as numerical values were not provided in the paper. ⮥ = gradual increase in temperature was applied in the HS group rather than static high-temperature exposure. ^a^ Cleavage rate decreased only with 42 °C exposure. At 41 °C, blastocyst rate decreased only after 12 h of exposure. ^b^ Within the study, one experiment observed a decrease in blastocyst rate, while another did not. ^c^ Analysis of embryo production was based on 12-h exposure. ^d^ Blastocyst rate decreased only when HS was done for 24 h at 41.5 °C. However, at 41.0 °C for 20 h, blastocyst rate was increased. ^e^ Analysis of embryo production was based on 1- and 4-h exposure. One-hour exposure did not affect blastocyst rate. ^f^ Fertilisation, cleavage, and blastocyst rate decreased only when heat stress took place between 12 and 22 h. ^g^ Cleavage and blastocyst rate only decreased at 40.5 °C.

Several studies utilising nuclear staining techniques to evaluate the maturation status of oocytes have reported a decreased percentage of oocytes reaching the MII stage when exposed to elevated temperatures. Specifically, these studies observed significant reductions in MII stage oocyte development at temperatures ranging from 39.5 to 41.5 °C. The exposure times in these experiments varied, spanning from as short as 3 h to as long as 24 h [61,122,124,136,143,145,146,148,156,166,167,168,169]. Still, some studies found that heat stress at 41 °C for 12 [139,147,161] or 24 h [170], or at 40.5 °C for 1–4 h [148] during IVM did not affect the percentage of MII oocytes. Similarly, contrasting results have been reported on the proportion of in vitro-matured MII oocytes derived from GV-stage oocytes exposed to elevated temperatures in vivo [60,72]. Interestingly, a model of heat stress during the first 12 h of IVM has shown accelerated oocyte nuclear maturation, leading to the fertilisation of an aged oocyte [129,142]. This hastening of oocyte maturation has been associated with altered temporal expression of *IL6* (promoter of cell differentiation) in oocytes [162]. In this in vitro model, early fertilisation of heat-stressed oocytes has been observed to partially improve blastocyst formation [137].

The successful maturation of the oocyte relies on coordinated communication with surrounding granulosa cells. Mural granulosa cells line the follicle wall and secrete hormones and growth factors crucial for oocyte maturation. Cumulus cells, a specialised subset of granulosa cells, are in direct contact with the oocyte, forming the cumulus–oocyte complex. Cumulus cells offer direct communication via gap junctions to provide metabolic support and mediate hormonal signals, especially during the LH surge that triggers the resumption of meiosis in the oocyte [91,171,172]. In vivo exposure models of heat stress have documented altered gene expression in granulosa cells. For instance, mRNA microarray analysis showed that granulosa cells of dominant follicles from Holstein cows exposed to 96 h of heat stress in an environmental chamber displayed upregulation of enzymes involved in G-protein coupled signalling, members of the solute carrier family, and follistatin, while exhibiting no changes in stress response genes, including apoptosis and heat shock proteins [173]. Similarly, RNA-sequencing analysis of dominant follicles’ cumulus cells from Holstein cows exposed in vivo to heat stress in a climate chamber for 12 h around the time of the LH surge did not affect genes associated with oxidative stress or apoptosis, but impacted genes involved in cell junctions, plasma membrane rafts, and cell cycle regulation [174]. At the oocyte level, an in vitro model involving a 12-h exposure to 41 °C indicated that most of the genes affected by heat stress were downregulated. These genes were predominantly associated with the electron transport chain and oxidative phosphorylation, both crucial for mitochondrial function [151]. However, a shorter exposure of 6 h at the same temperature did not affect the expression of genes involved in heat shock response (*HSPB11*, *HSP90AA1*, *HSPA1A*), antioxidant activity (*MnSOD*, *GPX1*), glucose metabolism (*G6PD*), and regulation of cell cycle (*CCNB1*) [159].

**Figure 1:**
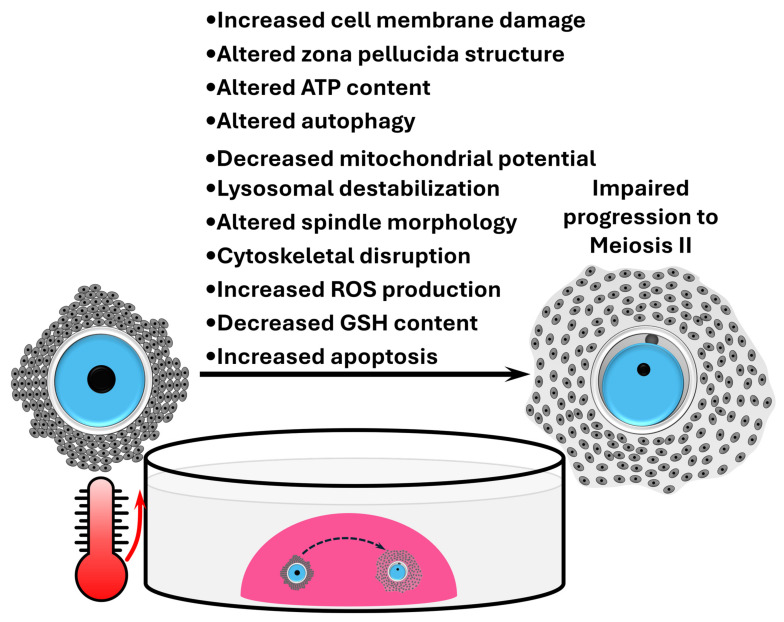
In vitro oocyte maturation under heat stress can result in several cellular and molecular alterations in oocytes. Model generated from cattle in vitro maturation models [122,124,132,135,136,138,143,144,145,146,147,150,151,156,158,160,165,167,170,177,178,179,180].

Cumulus–oocyte complexes as functional units are also responsive to thermal stress. Accordingly, 23 h of IVM at 40.5 °C can drastically alter the amino acid metabolism of COCs [166], while exposure to 41 °C for the first 6 [175] or 12 h [176] of a 24 h IVM cycle can increase progesterone production from cumulus cells. The integrity of gap junctions of COCs was also found to be affected when maturation occurred at 41 °C for 4 h [175]. Heat stress at 42 °C can disrupt oocyte protein synthesis during IVM of COCs, with this effect appearing more pronounced in the absence of cumulus cells, thus highlighting the protective role of cumulus cells during extreme thermal stress (Edwards and Hansen 1996). Considering the oocyte separately, several molecular and cellular variables indicative of oocyte quality have been reported to be affected by heat stress during IVM (Figure 1). However, these oocyte alterations acquired during IVM do not always have detrimental repercussions for embryo quality. For example, IVM at 41 °C for 12 h increased the percentage of oocytes showing apoptosis and high caspase activity, and inhibition of group II caspases in oocytes diminished the effects of heat stress on blastocyst formation. However, apoptosis levels were not affected in the resultant embryos, which showed an increase in total cell number [135]. Moreover, variables of oocyte quality do not consistently respond to heat stress. For instance, ROS levels and mitochondrial potential were unaffected when COCs were matured at 41 °C for 14 h [154] and at 41.5 °C for 22 h [124], respectively. Apoptosis levels were not affected either in oocytes matured at 41 °C for 12 h [139]. Similarly, while oocytes matured at 41 °C showed increased caspase activity after 12 h of IVM [135], this effect was not observed after 14 h of IVM [146]. Likewise, after 12 h of exposure to 41 °C during IVM, significant cell membrane damage was detected [165], whereas no such damage was evident after a 22-h IVM period at the same temperature [140]. Contradictory results also exist, with some studies reporting an increase in oocyte adenosine triphosphate (ATP) content [170,177] and others observing a decrease [165] under identical IVM heat stress conditions (i.e., 12-h IVM at 41 °C). Moreover, the autophagy marker LC3 in oocytes matured under 41 °C for 16 h and assessed via Western blot showed an increase [158], whereas a decrease was observed when oocytes underwent IVM at the same temperature for 6 h and analysed using immunofluorescence [177]. Whether this difference stems from the duration of thermal stress or the methodology employed to measure LC3 levels remains to be determined.

### 3.2. Cellular and Molecular Alterations in Bovine Blastocyst Derived from Oocytes Exposed to Heat Stress during In Vitro Maturation

The gene expression of blastocysts derived from COCs in vitro matured under heat stress has been also investigated in several studies. Pavani et al. [61] reported that blastocyst from oocytes cultured during IVM at 39.5 or 40.5 °C for 24 h showed downregulation of the heat shock protein *HSPA14* and upregulation of genes associated with regulation of methylation (*DNMT1*) and intercellular adhesion (*CDH1*). However, *DNMT1* expression was either not altered [160] or downregulated [165] when blastocysts were derived from oocytes exposed to 41 °C during IVM for 12 h. In another investigation, blastocysts from heat-stressed oocytes at 41 °C for 12 h expressed low levels of transcripts for the cell energy production gene *ATP1A1*, while genes linked to heat stress response (*HSP70.1*), antioxidant activity (*PRDX1*), and cell water transport (*AQP3*) were not affected [153]. With the same duration of IVM but at an increased temperature (41.5 °C), the same research group observed that produced blastocysts exhibited low expression of the heat shock protein *HSP40*, while transcripts for heat stress (*HSF1*) and pluripotency (*OCT4*) transcription factors, and other heat shock proteins, such as *HSP90AA1*, were not altered [152]. Interestingly, four-cell embryos derived from this model also displayed accumulation, as determined by immunofluorescence analysis, of trimethylation of histone3 lysine 9 (H3K9m3, a histone mark) and heterochromatin protein 1 (HP1, a transcriptional repressor), suggesting potential interference with embryonic genome activation [152].

The gene expression of heat shock protein *HSP90AA1* was not altered either in blastocysts from oocytes cultured for 6 h at 40.5 °C [157] or at 41 °C [159]. In the latter study, blastocysts also showed no changes in the expression of *HSPA1A* (heat shock protein), *BAX* (pro-apoptotic protein), *PTGS2* (enzyme involved in prostaglandin synthesis), *AKR1B1* (aldo-keto reductase enzyme), *SOD2* (antioxidant enzyme), *GLUT1* (glucose transporter), and *IGF2R* (growth factor). Nonetheless, the expression of *GPX1* (antioxidant enzyme) and *DNMT3A* (de novo DNA methyltransferase) was downregulated, while the expression of *PLAC8* (autophagy regulator) was upregulated [159]. Yet, the differential gene expression of *DNMT3A* and *PLAC8* in blastocysts from heat-challenged oocytes was not replicated in a subsequent study by the same research team [164]. Intriguingly, an upregulation rather than a downregulation of *DNMT3A* was present in blastocysts subjected to heat stress for 12 h at 41 °C [160]. However, using this same model, a recent paper confirmed the low expression of *DNMT3A* in blastocysts from heat-treated oocytes [165] previously reported by Stamperna et al. [159]. Adding to the confusion, the stable levels of *SOD2* and the decreased expression of *GPX1* observed in blastocysts derived from 6-h IVM at 41 °C [159], contrast with the lower *SOD2* expression and unchanged *GPX1* activity detected in blastocysts derived from 6-h IVM at 40.5 °C [157]. The latter study also documented an increased expression of heat shock protein *HSPA1A* and reduced levels of the antioxidant enzyme *CAT*, alongside unchanged expression of genes involved in cell proliferation (*AKT*), regulation of apoptosis (*XIAP*), and protection against oxidative stress (*SOD1, GPX4, NRF2*) [157]. Still, the previously reported stable expression profile of *GPX1*, *NRF2* [157], and *PRDX1* [153] in blastocysts produced under a 6–12 h IVM period at temperatures of 40.5–41 °C contrasts with the increased expression of these oxidative stress genes recently reported in blastocysts originating from oocytes matured during a 22-h period at 41 °C [181].

Other de novo DNA methyltransferases have been affected in blastocysts from oocytes subjected to 41 °C during IVM for 12 h, with a diminished expression of *DNMT3B* observed [160,165]. Applying the same IVM heat stress model, the upregulation of *BAX* and downregulation of *BCL2* were reported [156], which contrasts with the unaltered transcript activity of *BAX* documented in blastocyst resulting from oocytes experiencing a shorter period (i.e., 6 h) of the same heat stress conditions (i.e., 41 °C) during IVM [159]. Nevertheless, the increased abundance of *BAX* coupled with the low expression of *BCL2* has been also reported in blastocysts derived from a 22-h IVM period at 41 °C [181].

The above-discussed transcriptional activity of blastocysts suggests that the duration of heat stress during IVM, rather than subtle variations in temperature, may be more influential in altering gene expression dynamics in pre-implantation embryos from oocytes maturing under thermal stress in vitro. Regarding protein expression, it was recently documented that oocytes stressed for 12 h at 41 °C produced blastocysts with lower protein expression of several histones, including H1F0, H2A, H2B, and H4. Global DNA methylation was also altered in blastocysts derived from heat-stressed oocytes, as evidenced by the reduced levels of 5-methylcytosine (5mC) and 5-hydroxymethylcytosine (5hmC) [165]. In contrast, under identical IVM heat stress conditions, an increase in 5mC was reported in resultant blastocysts [160].

Other indicators of embryo quality, such as cell number, decreased in blastocysts derived from oocytes matured at temperatures between 41 and 41.5 °C for periods ranging from 6 to 24 h [119,122,131,134,139,144,146,147,161]. Similarly, an increase in apoptosis levels was observed in blastocysts from IVM models where oocytes were exposed to temperatures of 40.5–41.5 °C over periods lasting from 4 to 24 h [131,138,144,146,148,152]. Yet, under similar IVM heat stress conditions, no significant effects on cell number [89,129,138,140,152,153,157,166] and apoptosis [89,135,140,153] have been reported. In an IVM model where the temperature was gradually increased from 38.5 to 40.5 °C over a 6-h period, a decrease in both transcript and protein expression of interferon tau (IFNT) was reported in the resultant blastocysts. The enhanced production of IFNT was not associated with the number of cells expressing CDX2, a transcription factor involved in the cell lineage specification of the trophectoderm in blastocysts. Additionally, in this model, the resultant blastocysts displayed elevated ROS production [157]. Finally, although a temperature of 41 °C during IVM appears to alter ATP levels in oocytes [151,165], the ATP content in the resultant blastocysts remains unchanged [151].

### 3.3. When Exactly Is Oocyte Developmental Competence Impaired by Heat Stress during In Vitro Maturation in Cattle?

Several exposure times and temperatures have been used in IVEP models to assess the consequences of thermal stress during oocyte maturation in cattle (Table 1). Exposing bovine COCs to 41 °C during the first 6 h of IVM was sufficient to induce a decrease in the percentage of oocytes reaching metaphase II. This detrimental effect on oocyte maturation was exacerbated when the thermal challenge was sustained for up to 22 h. However, a decrease in blastocyst formation and hatching began to be observed after 12 h of thermal stress exposure [122]. Indeed, with this model (41 °C during the first 12 h of IVM), impaired blastocyst production rates have been reported by research groups from several countries including Brazil [147], China [165], Iran [156], Uruguay [122], and the USA [135,151]. Exposure of oocytes to 41 °C during the first 4 or 6 h of IVM did not affect the blastocyst rate [162]. However, other studies reported a decrease in blastocyst production using a 6-h period at the same [159,163,164] or slightly lower (i.e., 40.5 °C) temperature [157].

Exposing matured oocytes to a 4-h period at 42 °C following 20 h of IVM at 38.5 was sufficient to decrease blastocyst formation and their cell number in the trophectoderm, suggesting that a relatively short, abrupt increase in temperature can be detrimental for early embryo development [179]. Similarly, a 4-h exposure to 40.5 °C after 18 h of IVM resulted in decreased blastocyst formation and increased apoptosis levels [148]. This indicates that the late stages of oocyte maturation are also susceptible to thermal stress, although exposures lasting 1–2 h do not significantly affect blastocyst formation [179], unless IVM is conducted at 43 °C [182]. Similarly, a 1-h exposure of bovine oocytes to 41.5 °C at 8 h of IVM was sufficient to increase their protein expression of HSP70, but it did not affect blastocyst formation [149].

Hence, current evidence suggests that the in vitro maturation process is vulnerable to thermal stress between 40.5 °C and 42 °C in cattle. However, in a 22–24-h IVM protocol, at least 6 h of heat stress exposure at the beginning of IVM is required to affect oocyte competence, whereas at later stages of maturation, a minimum of 4 h of heat stress is necessary to impair the ability of the oocyte to become a blastocyst.

## 4. Impact of Heat Stress during the Fertilisation Process

After reaching metaphase II, the oocyte is picked up by motile cilia in the infundibulum and transported to the ampulla of the oviduct, ready to be fertilised by the sperm [183]. Once spermatozoa are deposited in the female reproductive tract via natural mating (vaginal deposition) or artificial insemination (usually intrauterine insemination) they travel to the oviduct. Research in cattle has shown that spermatozoa can reach the oviducts within the first hour after artificial insemination in the uterine body [184], passing through the uterotubal junction and establishing a so-called sperm reservoir in the isthmus, the initial segment of the oviduct [185,186]. During spermatozoa transit through the reproductive tract, a selection process occurs, including inside the oviduct, where spermatozoa with high DNA integrity that manage to cross the first selection barriers (e.g., vaginal pH, cervical mucus) are selected. Once in the isthmus, spermatozoa undergo capacitation, enabling them to acquire hyperactive motility that allows them to detach from the oviduct and move towards the site of fertilisation in the ampulla of the oviduct [185]. The fertilisation process involves a series of cellular and molecular events, including the acrosome reaction of the sperm, which enables the sperm to penetrate the zona pellucida and bind to the cell membrane of the oocyte [187]. After the sperm enters the oocyte, intracellular calcium oscillations are triggered, promoting oocyte activation. This leads to the resumption of meiosis II and activates mechanisms to prevent polyspermy, such as membrane depolarisation, elimination of the Juno protein (essential during sperm–egg fusion), and release of cortical granules that induce the hardening of the zona pellucida [186,188,189,190,191]. Sperm penetration results in the completion of the second meiotic division, culminating in the formation of female and male haploid pronuclei and the extrusion of the second polar body [186,192].

Indirect observations suggest that heat stress might impact the fertilisation process in vivo in cattle, as inferred from the increased pregnancy rates in Holstein heifers that lowered their rectal temperature from 39.9 °C to 38.7 °C through cooling applied from 2 h before to 2 h after artificial insemination [193]. However, the analysis of heat stress on sperm biology inside the reproductive tract will require an acute experimental induction of heat stress exclusively during the time of artificial insemination, followed by the collection of reproductive tracts to evaluate sperm populations within the different sections of the reproductive tract and the status and quality of resultant presumptive zygotes. To the best of the authors’ knowledge, there are no in vivo studies addressing the effect of heat stress on the fertilisation process per se in cattle. As such, the current knowledge on this subject in cattle has been generated in laboratory-based studies using in vitro fertilisation models.

In previous research, it was found that blastocyst production decreased when IVF took place for 8 h at 41.5 °C, but not at 40 °C [194], and when temperatures were gradually increased from 39.5 °C to 41 °C during the first 12 h, then lowered to 40 °C for the next 12 h of a 24 h IVF protocol, a significant reduction in cleavage rate was observed [195]. In the 1980s, it was reported that exposure to 40 °C for 22 h increased the occurrence of the acrosome reaction [196], a process essential for fertilisation [187]; this suggested that a decreased fertilisation rate may cause impaired embryo production following IVF under thermal stress. However, subsequent research found that when IVF was conducted for 6 h at 40 °C or 41 °C, the level of polyspermy was not clearly affected [197]. Nevertheless, a decrease in cleavage rate and blastocyst formation was noted, and analysis of oocytes fertilised at 40 °C revealed that this detrimental effect on embryo development was associated with increased production of ROS, elevated expression of heat shock protein *HSPA1A*, and downregulation of a gene involved in the prevention of polyspermy (*UCHL1*). Despite these effects, the cell number of the resultant blastocysts remained unchanged [197]. Similarly, oocytes undergoing IVF at 40.5 °C for 6 h showed reduced rates of embryo cleavage and blastocyst production [157,198]. The compromised embryonic development was connected to an increased presence of cathepsin B at the four-cell stage [198], a lysosomal cysteine protease involved in intracellular proteolysis within the lysosome, alongside its roles in apoptosis and autophagy [199]. The resultant blastocysts did not show alterations in cell number [157,198] or apoptosis [198], but produced less interferon tau and displayed increased expression of genes linked with oxidative stress regulation (*GPX1*) and heat shock response (HSPA1A), along with downregulation of another oxidative stress response gene (*CAT*) [157]. A recent study also reported a diminished blastocyst production when IVF was conducted at 41 °C for 6 h; however, interestingly, an increase in blastocyst yield was observed when IVF was performed at 39.8 °C [177]. An important question in these in vitro models is whether the effect of heat stress impacts the oocyte, sperm, or both. In vitro data indicated that exposure to temperatures of 40–42 °C for 4 h can decrease bovine sperm motility [200,201], yet using these heat-stressed sperm for IVF did not affect the rate of polyspermy and blastocyst formation [201]. Likewise, exposing sperm to 41 °C for 4 h did not affect sperm cell membrane damage, and there was no impact on blastocyst production following IVF. Intriguingly, sperm cell membrane damage was observed during a 6-h IVF under thermal stress at 40 °C. The reason for this discrepancy is unknown, but it might be associated with the duration of exposure (i.e., 4 versus 6 h) [197]. Hence, the in vitro evidence suggests that during IVF, the detrimental effect of heat stress on embryo development is mainly exerted via the oocyte.

It is unknown whether bovine sperm motility is affected in vivo by heat stress, but indirect information suggests that this might not be the case, as indicated by the presence of accessory sperm on unfertilised oocytes collected 5 days after ovulation during summer in putative heat-stressed dairy cows [202]. The IVF data discussed above also suggest that heat stress does not appear to substantially affect post-ejaculated sperm in cattle. Nevertheless, it is well documented that spermatogenesis can be affected by heat stress in mammals, including cattle [203,204]. As reviewed by Morrell [205], the effects of season on sperm quality in cattle show contrasting results, which could be attributed to differences in THI levels across studies during hot months or variations in the timing of sperm examination following heat stress. Moreover, the absence of identification of heat-tolerant bulls may impact seasonal analysis, as certain bulls can consistently produce high-quality spermatozoa year-round, irrespective of temperature [206]. Still, studies have consistently demonstrated that seasonal exposure of bulls to heat stress can lead to the production of sperm with impaired functionality, negatively affecting IVEP [207,208,209]. Experimental induction of heat stress through testicular insulation for 48 h has also shown that bulls subjected to thermal stress produced sperm exhibiting impaired pronuclear formation, which was associated with difficulties in achieving sperm head decondensation [210]. However, the impact on the quality of the resulting embryos appears to be breed-specific, as the cell count decreased and apoptosis levels increased in blastocysts produced with heat-stressed sperm from Belgian Blue bulls [209], while both parameters remained unchanged in Holstein Friesian bulls [208]. Additionally, gene expression in blastocysts was not affected [208]. The diminished embryo production from sperm of heat-stressed bulls emphasises the importance for companies marketing cattle semen to consider the potential consequences of collecting semen during periods of heat stress.

## 5. Impact of Heat Stress during Pre-Implantation Embryo Development

Following fertilisation, the newly formed zygote will go through a series of cell divisions to achieve the blastocyst stage during the pre-implantation period, along with several molecular and cellular milestones including epigenetic reprogramming, embryonic genome activation, and cell lineage specification to allow differentiation of cells in the inner cell mass (ICM) and trophectoderm (TE) [211]. The cells of the TE surround the ICM and will form the placenta, while the ICM comprises the epiblast and primitive endoderm, which will form the foetus and yolk sac, respectively [212]. As such, the pre-implantation embryo is highly susceptible to environmental challenges that can result in embryo mortality [211,213].

The effect of in vivo heat stress on bovine pre-implantation embryo development has been explored using superovulated cattle. Research in the late 1980s demonstrated that embryo degeneration increased in Holstein heifers kept in a climate-controlled room with cycles of 16 h at 30 °C and 8 h at 42 °C from day 1 to day 7 after artificial fertilisation [214]. Comparing shaded and non-shaded Holstein cows in the USA, Ealy et al. [87] found that blastocyst production was reduced in Holstein cows exposed to heat stress (with a rectal temperature of 40.9–41.7 °C) on day 1 of pregnancy. This detrimental effect on embryonic development, however, was not observed when the cows experienced heat stress on days 3–7 of pregnancy. In Brazil, it was reported that embryo production in both cows and heifers declined during the hot season (13–37 °C) compared to the cool (11–17 °C) season [215]. However, seasonal effects are contradictory in this regard, as embryo production was not different between the cold (THI 71) and the hot (THI 74) seasons in Holstein Friesian cows raised in Brazil, but it decreased during EL Niño-related heat waves (THI 80) during 1997–1998 [216]. Similarly, no differences in embryo production from superovulated cows were found when comparing the dry (average 12 °C) and rainy (average 31 °C) seasons in Mexico. Intriguingly, the number of apoptotic cells in good and poor-quality embryos produced was decreased during the season with the higher temperatures (i.e., rainy season) [217]. In another study conducted in a different region of Mexico, which also compared the dry (11–22 °C) with rainy (16–27 °C) seasons, no discernible differences were observed in the production of transferable embryos [218]. However, these seasonal studies do not allow for delineating the direct effects of heat stress on pre-implantation embryo development. To this end, IVEP studies have provided a good model to identify specific developmental stages affected by heat stress during the pre-implantation period.

Bovine in vitro studies examining the effects of heat shock have shown that exposing pre-implantation embryos to high temperatures between 39.5 and 42 °C significantly decreases the rate of blastocyst formation. The temperature range of 40 to 41 °C is most commonly used in these experiments, with exposure durations varying from as short as 6 h to as long as 24 h. The early cleavage stages, particularly up to the 8–16-cell stage, are reported to be especially vulnerable to heat stress, compared to the morula formation period in bovine embryos around days 4–5 after IVF (with IVF designated as day 0) (Table 2). It has been proposed that the enhancement of thermotolerance during early development is associated with the acquisition of molecular traits that confer thermal resilience as the embryo progresses. Additionally, the increasing cell number in the developing embryo enables it to endure the loss of certain cells without impairing its growth [120]. But the method of heat exposure may impact how early embryos respond to heat stress. For example, abrupt static exposure of one-cell embryos to 40.5 °C for 10 [219], 12, or 24 h [220] damaged their capacity to form blastocysts, while a gradual increase to the same temperature over a 19-h period did not affect the blastocyst formation rate [194]. Still, these in vitro models do not consistently show detrimental effects of heat stress on pre-implantation embryos. For instance, it was reported that two-cell embryos had a reduced ability to reach the blastocyst stage when cultured at 41 °C for 9 or 12 h, but not for 3 or 6 h at the same temperature [194]. However, a subsequent study from the same group found that culturing for just 6 h at 41 °C was sufficient to impair the potential of two-cell embryos to form a blastocyst [221]. In the same vein, exposing embryos on day 4 after IVF—when they typically have more than eight cells and are in the process of forming a morula—to 41 °C for 6 h has been reported to decrease blastocyst production in some studies [222,223], while other studies [224] found no such effect. These contradictory findings could be related to batch variations during IVEP cycles and differences in culture conditions, such as the type of supplementation in the culture medium.

### 5.1. Molecular and Cellular Response of Pre-Implantation Embryos to Heat Stress

Several molecular alterations around the time EGA have been linked with the compromised ability of early embryos to achieve the blastocyst stage following thermal stress at the one-cell stage. For instance, impaired blastocyst formation arising from heat-stressing one-cell embryos for 6 h at 41.5 °C was associated with increased ROS production and reduced levels of glutathione (GSH) at the 8- to 16-cell stage [231]. Both ROS and GSH play a significant role in modulating the response of pre-implantation embryos to heat stress in vitro [239]. In a similar manner, one-cell embryos cultured for 20 h at 40 °C showed a decreased ability to form blastocysts, associated with increased expression of cathepsin B at the four-cell stage [198]. The reduced blastocyst formation rate has also been linked to low intracellular calcium levels in 1–2-cell embryos exposed to 40.5 °C for 10 h, starting 20 h after IVF [219]. Electron microscopy studies have also revealed that exposing two-cell embryos to 41 °C for 6 h caused damage to the cytoskeleton and mitochondria, hindering their ability to reach the blastocyst stage [221]. When thermal stress occurs during the later stages of embryonic development, comparable outcomes have been noted. For example, the decreased blastocyst formation observed in 4–8-cell embryos exposed to 41–41.5 °C for 6 h was associated with an increase in ROS levels in the 8–16-cell stage [224,231]. Likewise, embryos exposed twice for 10 h each at 40.5 °C around the time of morula formation (i.e., days 4–5 after IVF) showed a decreased mitochondrial membrane potential at the early blastocyst stage (i.e., day 6 after IVF) [235].

The bovine pre-implantation embryos seem to increase protein synthesis in response to heat stress, including heat shock proteins [121,240]. Gene expression analysis indicated that upregulation of heat shock proteins is evident at the two-cell, four-cell, and morula stages [126,241]. However, a transcriptomic analysis suggested that the resistance of bovine morulae to heat stress does not primarily rely on the upregulation of heat shock proteins and antioxidant genes but rather on their ability to produce cellular antioxidants to prevent free radical accumulation and to remove denatured proteins, presumably through the regulation of genes encoding proteins that activate Ubiquitin C [127]. Interestingly, unlike two-cell embryos [242], around the early morula stage, a moderate presence of apoptosis is believed to promote embryo survival following thermal stress in vitro [225]. Accordingly, there is evidence that morulae can acquire further resistance to heat shock via mild exposure to heat stress associated with increased protein expression of HSPA. In this model, embryos at the morula stage were subjected to six 1-h heating sessions, with each session starting at 38.5 °C and reaching 40.5 °C, with temperature increases of 0.5 °C every 7–8 min. The temperature then immediately decreased back to 38.5 °C at the same temperature interval and time rate. When these heat-treated morulae were exposed to 41 °C for 2 h, developmental arrest was decreased compared to control counterparts [243]. In this regard, research has shown that morulae exposed to 41 °C for 9 or 15 h increase their levels of apoptosis and have a lower cell number, which is associated with a diminished ability to reach the blastocyst stage [228,244,245]. However, the alteration in cell number was not observed in another experiment by the same group using the 9-h 41 °C protocol [232]. The gene expression profile of the resultant blastocysts seems to be affected differently depending on the embryonic stage at which heat shock is applied and the duration of exposure. Exposure of 2–4-cell embryos to 41 °C for 24 h resulted in the production of blastocysts with increased expression of *HSPA1A*, *PLAC8*, *ATP1A1*, *AKR1B1*, *GSTP1*, *HSF1*, *PTGS2,* and *TLR4*, while decreasing the expression of *BCL2* and *DNMT3A*. Expression of *HSP90AA1*, *GPX1*, *TLR2*, *BAX,* and *IGF1* was not affected [238]. Nellore and Jersey embryos exposed to 41 °C for 6 h at the 8–16-cell/early morulae stage developed into blastocysts with low expression of *PLAC8*, *HSF1*, and *CDX2*. However, *COX2* (regulator of inflammation, cell proliferation, and apoptosis) was only downregulated in Holstein blastocysts [236]. Blastocysts exposed to 41 °C for 6 h showed increased expression of *BAX* and *HSPA1A*, while levels of *IFNT2* (gene involved in maternal recognition of pregnancy), *POU5F1*, *CDX2*, *PLAC8*, *SOD2*, and *COX2* remained unchanged [237]. The unaffected gene expression of *IFNT2* contradicts the lower transcript levels of *IFNT2* and the protein expression of IFNT documented in blastocysts derived from one-cell embryos that were heat-stressed at 40.5 °C for 6 h [157]. Moreover, the mixed transcript repertoire reported in the aforementioned studies indicates that genes such as *PLAC8* (an autophagy regulator) exhibited varying responses at the blastocyst stage, being either unaffected, downregulated, or upregulated. This variability offers no clear insight into the potential autophagy activity in blastocysts resulting from heat stress during early divisions. Other genes relevant to pre-implantation embryo development (e.g., *CDX2*) also showed inconsistent expression patterns in response to heat stress, reflecting the complexity of gene regulation during this crucial period. Similarly, cellular variables such as reduction in cell number and increased apoptosis are not always affected in in vitro heat stress models (Table 2). Furthermore, some studies have observed that following embryo transfer, there was no difference in pregnancy rates between heat-stressed and control embryos [236].

### 5.2. Are the Detrimental Effects of Heat Stress on Pre-Implantation Embryos Caused by Altered Function of the Reproductive Tract?

An important consideration is whether the effect of heat stress on the pre-implantation embryo could be mediated through the tissues of the reproductive tract. Bovine endometrial cells exposed to 43 °C altered their protein secretion [246,247]. However, in a more relevant model, protein synthesis remained unchanged in bovine uterine cells cultured at 41 °C for 9 h [223]. Increased prostaglandin secretion was reported in bovine endometrial tissue exposed to 43 °C for 18 h [248]. This finding was confirmed in a recent study by Sakai et al. [249], where endometrial stromal cells increased their production of prostaglandin (PG) E2 and PGF2α after exposure to heat stress at 40.5 °C for 10 h, twice, with a 14-h interval between exposures The increased production of PGF2α in the endometrium was linked to a heightened ability of tumour necrosis factor (TNF) α to stimulate PGF2α synthesis, along with a diminished effectiveness of interferon tau (IFNT) to suppress PGF2α production [250]. In contrast, culturing a mix of bovine endometrial epithelial and stromal cells for either 24 h or 8 days at 40.4 °C resulted in a decreased production of PGF2α [251]. The reasons for these discrepancies are unclear, but it is possible that factors such as the duration of culture, specific cellular interactions between the epithelial and stromal cells, the stage of the oestrous cycle used for sample collection, or other environmental conditions during culturing may have influenced the regulation of PGF2α production in vitro. Nevertheless, the altered prostaglandin secretion induced by heat stress is more likely to impact oestrous cyclicity rather than directly affecting the embryo. This disruption in hormone levels could interfere with the regular reproductive cycle, potentially leading to issues with pre-implantation embryo survival.

Using the in vitro model of Sakai et al. [249], but adding a 12-h lipopolysaccharide (LPS) challenge under heat stress (i.e., 40.5 °C), it was shown that heat-stressed endometrial stromal cells increased their gene expression of inflammatory cytokines, resulting in disrupted recruitment of macrophages [252,253]. Similarly, endometrial epithelial cells challenged with LPS for 24 h under 41 °C were shown to upregulate the transcript expression of proinflammatory mediators [254]. In addition, exposing bovine endometrial epithelial cells to 40.5 °C for 12 h led to the activation of an oxidative stress response [255]. The intensified inflammatory response associated with heat stress, although speculative, may interfere with the ability of the endometrium to produce histotroph—the nutrient-rich fluid from endometrial glands that nourishes the embryo before implantation [256]—and, in turn, compromise embryo viability.

Indeed, experimentally induced endometrial inflammation in cattle has been shown to alter histotroph metabolites, which are crucial for supporting early embryonic development [257]. The inflammation-induced changes in metabolite composition, along with the reduced uterine blood flow caused by heat stress [258], could disrupt the nutritional microenvironment in the uterus necessary for embryo viability, potentially leading to impaired implantation.

Embryo transfer models have also suggested that heat stress may alter uterine function. For example, the proportion of dairy cows unable to display a secretory peak of endometrial epidermal growth factor (EGF), which is associated with sound fertility [259], was higher during hot months [260]. This lack of an EGF peak was linked to decreased pregnancy rates following embryo transfer [260]. Similarly, the conception rate after embryo transfer declined in Holstein cows, but not in heifers, when exposed to temperatures exceeding 25 °C and a THI above 75 [261]. Likewise, exposing dairy heifers to a THI ranging from 73 to 78 on the day of embryo transfer (ET) or seven days after ET did not affect pregnancy rates [262]. This suggests that heat stress further aggravates the already compromised reproductive tract microenvironment of lactating dairy cows [263], which could cause adverse effects on pre-implantation embryo survival. At the oviduct level, it has been reported that protein synthesis remained unaffected in bovine oviductal cultures exposed to 41 °C for 9 h [223]. Similarly, recent in vivo studies demonstrated that Simmental cows exposed to a THI of 72 or higher did not experience significant changes in the oviductal proteome [264]. However, it is important to note that in vivo heat stress models should identify animals actually experiencing heat stress, rather than assuming they are under stress solely based on THI values. This distinction is particularly relevant given the presence of heat-tolerant cattle [265], including dairy cattle [266,267].

## 6. Impact of Breed on Oocyte and Pre-Implantation Embryo Thermotolerance in Cattle

It is well documented that some cattle breeds are better adapted to handling heat stress [268,269]. Bos indicus breeds are considered well-adapted cattle that have shown a better performance in terms of IVEP in countries with tropical and subtropical climates [270,271]. Accordingly, in an OPU-IVEP study comparing *Bos taurus* (Angus and Holstein) with *Bos indicus* (Brahman) cattle, it was reported that oocytes collected during the hot season produced fewer blastocysts in *Bos taurus* but not in *Bos indicus* cattle [55]. Similarly, a 6-h in vitro exposure to 41 °C around the time of morula formation (i.e., day 4 after IVF) decreased blastocyst formation in Holstein embryos, whereas Brahman embryos exhibited an increase in IVEP [222]. However, while less pronounced than in *Bos Taurus* embryos (i.e., Holstein and Angus), a decrease in blastocyst formation was observed in Brahman embryos subjected to the same in vitro heat stress conditions [223,227]. Indeed, other tropically adapted breeds, such as Nellore and Romosinuano, have also shown decreased embryo production when challenged at day 4 post-IVF with 41 °C for 12 [272] or 6 h [227], respectively. Similarly, decreased blastocyst production was reported in Brazil with oocytes from Zebu cattle that were in vitro matured at 40 °C, even though the expression of *HSP70* and *HSP90* in these oocytes remained unaffected [130]. Likewise, GV-stage oocytes from Gir cows that were experimentally exposed to temperatures ranging from 30 to 38 °C for 28 days resulted in decreased IVEP [73]. A higher expression of the heat shock protein *HSP70* has been reported in GV-stage oocytes from Holstein compared to Gyr cows, partially accounting for the thermotolerance present in Gyr cattle. Still, following embryo transfer, there was no difference in pregnancy and calving rate between the two breeds [273]. A microarray analysis has also revealed transcript differences in in vitro matured oocytes between Holstein and Nellore cattle, including genes involved in embryonic development, cell death and survival, cell cycle, and free radical scavenging. Interestingly, oocyte gene expression was not altered significantly between breeds when oocytes were exposed to heat stress in vitro [274].

Breed differences in oocyte thermotolerance have also been observed in *Bos taurus* cattle. For instance, research indicates that oocytes from Limousin cows cultured at 41 °C for 6 h experienced significantly greater impairment in blastocyst formation compared to those from Holstein cattle. This difference in thermotolerance was associated with the upregulation of the heat shock protein gene *HSP90AA1* in matured oocytes from Holsteins. Additionally, the resultant blastocysts from these two breeds exhibited distinct gene expression profiles in response to the thermal challenge. Specifically, blastocysts derived from Holstein cattle showed increased expression of the gene *PLAC8*, which is linked to embryo development and implantation. In contrast, embryos from Limousin cattle demonstrated decreased expression of key genes such as *HSP90AA1*, *DNMT3A*, and *SOD2*, which are crucial for cellular stress response, DNA methylation, and oxidative stress protection, respectively [163].

Comparing Holstein, Angus, and Braham sperm, it was found that heat stress affected sperm motility regardless of breed, suggesting that the genetic composition does not seem to play a significant role in heat stress protection at the sperm level. [200]. However, studies examining combinations of oocytes and sperm from *Bos indicus* and *Bos taurus* breeds found that in vitro heat stress during pre-implantation embryo development reduced blastocyst production, irrespective of the sperm source used [222,272]. In contrast, Eberhardt et al. [275] reported that using *Bos indicus* sperm could mitigate the effects of in vitro heat stress on Holstein oocytes. However, this study was criticised for the low number of bulls tested per breed [271]. Nonetheless, the prevailing consensus in IVEP bovine models suggests that the resilience of the pre-implantation embryo to thermal stress is predominantly determined by the oocyte, rather than the sperm [271]. Research using somatic nuclear transfer models has indicated that the oocyte cytoplasm, rather than the nucleus (i.e., donor cell), is primarily susceptible to heat stress, leading to a reduction in blastocyst formation. This negative impact was evident when the ooplasm was obtained from Holstein cattle. In contrast, this detrimental effect was not observed when the ooplasm originated from Taiwan Yellow cattle, a heat-tolerant Bos indicus breed [276]. The cloned offspring derived from reconstructed oocytes using ooplasm from Taiwan Yellow cattle, in comparison to ooplasm from Holstein cattle, exhibited somatic cells (i.e., ear cells) with lower protein expression of pro-apoptotic regulators (BAX, AIF, CYTOCHROME C, CASPASE-3, -8, and -9), coupled with increased expression of anti-apoptotic (BCL-2) and heat shock (HSP27, HSP70) proteins [277]. The same group produced calves using spindle transfer technology, in which spindle–chromosomal complexes from Holstein cattle were transferred into ooplasm from Taiwan Yellow cattle, followed by IVF with Holstein sperm [278]. The somatic cells from the spindle transfer offspring exhibited greater thermotolerance than those from Holstein cattle when subjected to an extreme thermal challenge at 42 °C for 12 h. Gene and protein analyses revealed that this thermotolerance was associated with lower pro-apoptotic activity and enhanced functions of anti-apoptotic, oxidative phosphorylation, and antioxidant pathways [279].

Still, this thermotolerance has its limits, as illustrated by the decrease in IVEP outcomes for *Bos indicus* breeds when their oocytes or embryos were exposed to heat stress in vitro [130,223,227,272]. Furthermore, natural in vivo exposure can also affect the reproductive physiology of *Bos indicus* breeds. For instance, Thai indigenous beef cows exposed to THI of 83 during the rainy season exhibited decreased pre-ovulatory follicle size and vascularity, along with reduced oestradiol concentrations during the pre-ovulatory period [102]. These findings suggest that even within heat-resistant breeds, there may be individuals with less marked thermotolerance.

## 7. How Relevant Are In Vitro Models to Elucidate the Impact of Thermal Stress on Oocyte Developmental Competence and Embryo Viability?

The majority of IVEP models of heat stress have been developed using rectal temperatures of cattle subjected to heat stress. It has been suggested that cows with a rectal temperature of 39.1 °C or higher can be considered to be experiencing heat stress [280], and that a rectal temperature of 39 °C is expected to impair fertility in cattle [11]. This contrasts with control animals showing a rectal temperature of 39 °C used to analyse the effect of heat stress on pre-implantation embryo development in the early 1990s [87]. Previous research reported that dairy cattle enduring heat stress at an ambient temperature of 30.5–34.7 °C (dry bulb temperature) in Florida, USA, presented rectal temperatures ranging from 40.9 to 41.7 °C [87], but a later study on the same region indicated that cows exposed to peak temperatures of 34 °C displayed average rectal temperatures of 40.5 °C [194]. Subsequent research in the same USA state showed that THI values ranging from 81 to 92 were associated with rectal temperatures of 40–41 °C, depending on the methodology used to calculate the THI values [11]. In Arizona, USA, Holstein and Brown Swiss cattle kept under THI of 81–82 maintained their average rectal temperatures below 40 °C [281]. Similarly, research conducted in Brazil with *Bos taurus*, *Bos indicus*, and their crosses has demonstrated that cattle experiencing heat stress, with maximum THI ranging from 79 to 87, did not exceed an average rectal temperature of 40 °C [282,283,284,285]. The rectal temperature of dairy cows in Saudi Arabia exposed to a THI of 84 remained below 40.5 °C [286]. During the summer in Tunisia, where the THI was 78, Holstein cows showed an increase in rectal temperature to 39.6 °C [287], similar to the rise to 39.1 °C in Holstein cows exposed to THI values of 72–78 in Italy [288]. In Egypt, dairy cows exposed to an average THI of 82 during the summer did not have rectal temperatures greater than 40.1 °C [107]. In Canada, during controlled natural ambient temperature exposure (i.e., not a seasonal comparison) to THI values above 80, rectal temperatures in dairy cattle remained below 39.5 °C [289]. Likewise, in China, the rectal temperature of dairy cows exposed to a THI of 80–86 stayed under 39.5 °C [290], and at a THI of 90, the maximum rectal temperature recorded was 40.8 °C [291]. Additionally, experimental studies using environmental chambers to mimic THI values of 76 [173], 83–86 [174,292], and 92 [73] reported average rectal temperatures ranging from 39.3 to 40.2 °C. Accordingly, multiple studies conducted over 40 years ago working with different models of heat stress in cattle reported maximum rectal temperatures of 40–40.2 °C, as reviewed by Gwazdauskas [293].

Vaginal temperature has been also used in in vitro models of heat stress [143]. In the 1950s, it was noted that the average vaginal temperature of dairy cows ranges from 38.5 to 38.6 °C during the oestrous cycle [294], a range similar to the 38.1–38.7 °C observed in more recent studies [98,295,296,297,298], with increases to around 39 °C observed at the time of oestrus [296,297,298]. However, maximum vaginal temperatures of up to 41.1 °C can be observed during oestrus [299], and during the early postpartum period (i.e., between days 2–10 postpartum), vaginal temperatures of 39.5 °C to 41.4 °C have been reported in dairy cows, which is suggested to be associated with inflammatory processes typically present during this physiological state [300]. Several studies have reported an increase in vaginal temperature associated with heat stress in cattle, with deviations in temperature similar to those observed in rectal temperature studies. For example, daily circadian increases from 39.7 to 40.7 °C during a hot period with a THI of 77 were observed in Holstein Friesian cows in Japan [98]. Also in Japan, vaginal temperature did not increase beyond 38.8 °C in Japanese Black cows during the summer when THI was 77 [298]. In Holstein cows during the first 10 days postpartum, a mean vaginal temperature of 39.6 °C, ranging from 38.8 to 40.4 °C, was recorded during a hot period with a THI of 74 in Germany [301]. In pregnant beef cattle, average vaginal temperatures remained below 40.5 °C during hot weather in the USA, with a THI ranging from 76 to 83 [302]. Similarly, in the same location, beef heifers with THI values of 84–86 did not exceed an average vaginal temperature of 40 °C [303]. In Brazil, Girolando cows (a crossbreed of Gir and Holstein cattle) exposed to a mean THI value of 75 displayed an average vagina temperature of 39.5 °C [304]. In a study conducted during the Australian summer, heat-susceptible beef cattle, identified by their pronounced panting, exhibited a body temperature of 38.9 °C, which was slightly higher than that of heat-tolerant heifers, whose vaginal temperature measured 38.6 °C [265]. Indeed, it is well established that cattle exhibit individual physiological responses to heat stress, with some animals showing better adaptation and coping mechanisms to manage heat stress than others [305,306,307,308]. More recently, in Holstein cows in Italy, an average vaginal temperature of 39.3 °C was observed during heat wave days in Italy with a mean THI value of 77 [309].

Hence, the majority of heat stress research in cattle suggests that rectal and vaginal temperatures do not usually surpass 41 °C, even when cattle experience severe heat stress. Nonetheless, maximum rectal temperatures of 41.7 °C [202] and vaginal readings of 41.4 °C [309] have been reported in lactating cows enduring heat stress. Noteworthy, in veterinary medicine, cattle suffering from heat stroke, rather than heat stress, often exhibit rectal temperatures above 41 °C [310]. As such, in vitro models using temperatures above 41 °C to examine the effect of thermal stress on oocyte developmental competence and embryo viability are of limited relevance for elucidating the direct effects of high temperatures on cellular and molecular variables in oocytes and embryos. By the same token, it is advisable to avoid using 39 °C in the control group instead of 38.5 °C to more accurately replicate in vivo conditions.

Still, the question remains as to whether physiological increases in rectal and vaginal temperature are reflected in ovarian follicles and the lumen of the oviducts and uterus. As discussed in recent reviews [311,312], the temperature in bovine pre-ovulatory follicles is approximately 1 °C lower than that of the ovarian stroma, uterine lumen, and rectum—a cooling effect observed consistently across several mammalian species, but considered crucial for ovulation and positively linked with pregnancy potential in dairy cattle and humans. Experiments in cattle have revealed that pre-ovulatory follicles need to reach temperatures in the range of 36.8–37.6 °C to achieve ovulation during hot weather, with THI values ranging from 72 to 86 [313,314,315]. However, no information is available on the quality of these ovulating oocytes [312]. This physiological cooling would be essential given the so-called higher oestrous-associated temperatures (HEAT) present around oestrus [299], which have been found to promote pregnancy success in a thermoneutral environment [316,317], presumably via changes in the intrafollicular metabolome during the preovulatory period [318]. Nevertheless, the ability to lower the temperature of the reproductive tract following HEAT could be key to successful reproduction, as a previous study reported that cooling of the cervix was associated with an increased likelihood of achieving pregnancy [319]. Albeit speculative, the temperature-dependent ovulation observed in cattle suggests that a failure to achieve sufficient cooling around ovulation, rather than an increase in follicle temperature, might be associated with the detrimental effects of heat stress on bovine oocyte competence. Still, it is unknown whether high ambient temperatures can sufficiently raise follicular fluid temperature to adversely affect oocyte competence, as seen in in vitro models. As a starting point to elucidate this issue, a bovine IVEP model could combine acute experimental induction of heat stress [174] in superstimulated cows to collect in vivo-matured oocytes via OPU [320], along with measurement of follicular fluid temperature [315] at the time of oocyte retrieval. These types of experiments are essential to corroborate that in vitro models accurately simulate the conditions of oocyte maturation during in vivo heat stress. From this perspective, current IVM models assume increased intrafollicular temperature during heat stress, raising questions about whether the detrimental effects on oocyte competence during maturation are due not to direct high temperature exposure, but rather to alterations in ovarian physiology. These could include decreased ovarian blood flow [101,102], which may disturb pre-ovulatory follicle cooling linked to successful ovulation [311,312,315], as well as oocyte competence, as demonstrated in women [321,322]. Heat stress-induced diminished ovarian blood flow may also disrupt the supply of essential nutrients, ions, and hormones necessary for oocyte maturation [323].

In vitro models addressing the effect of heat stress during the fertilisation process and pre-implantation embryo development have also relied on rectal or vaginal temperatures. Both rectal [317,324] and vaginal [296,297,299] temperatures increase during oestrus, suggesting that under natural mating conditions, the sperm enters the reproductive tract in a high-temperature microenvironment. Indeed, there is a temperature gradient in the bovine reproductive tract, with temperatures gradually increasing from the vagina towards the uterine horns [325]. The temperature in the lumen of the bovine uterus is slightly higher than the rectal temperature, around 0.2 °C [325,326], and research from the 1970s found that the temperature in the uterine lumen around the time of oestrus ranges from 38.3 to 38.6 °C, with an increase of 0.5 °C above 38.6 °C resulting in a 12% decrease in conception rate [326]. To the best of the authors’ knowledge, this is the only study showing this relationship, and it was not conducted under experimental heat stress conditions. Therefore, additional research is required to confirm these findings. Furthermore, although a positive correlation between uterine temperature and THI values was observed in Japanese Black cows, the increase in uterine horn temperature did not surpass 39 °C during summer when the THI was equal to or greater than 74 [327]. In this study, heat stress during summer increased uterine temperature by only 0.5 °C, suggesting that current in vitro models addressing thermal stress during pre-implantation embryo development do not accurately replicate the temperature conditions observed in vivo during heat stress. Nevertheless, monitoring uterine lumen temperature during in vivo heat stress should focus on more vulnerable physiological states and breeds, such as lactating Holstein cows, since heat-sensitive breeds and cows with high metabolic demands may potentially experience a more pronounced increase in uterine temperature. From this point of view, the possibility exists that the uterus might be able to maintain a relatively stable temperature during thermal strain, and that the detrimental effect of heat stress on embryo development could be related to the altered function of the uterine tissue rather than a direct thermal challenge to the embryo.

At the oviductal level, research in pigs has found that the uterus is approximately 1.5 °C warmer than the oviduct, a difference observed in sows but not in gilts [328]. For many years, based on previous studies in rabbits and pigs, it has been asserted that the caudal region of the isthmus is 1–2 °C cooler than the ampulla, and that this temperature gradient directs sperm towards the warmer site of fertilisation in the ampulla [311,312]. However, this finding has been recently disputed with experiments in mice [329] and pigs [328]. Currently, there are no available data on oviductal temperature in cattle, nor is there any information regarding a potential temperature difference between the uterus and oviduct. Consequently, it remains unknown how much the temperature varies in the oviduct during heat stress in cattle. Despite the technical challenges involved, it might be feasible to access and measure the temperature of the oviducts in live cattle using transvaginal endoscopy [330]. Hence, uncertainty remains regarding whether current in vitro bovine models accurately simulate the high thermal microenvironments present in the oviducts of heat-stressed cattle during fertilisation and early embryo development, as oviductal temperatures during heat stress have not been directly measured.

Although challenging, it will be relevant to obtain intrafollicular and intraluminal uterine and oviductal temperatures during periods of natural heat stress exposure in cattle, as this will help confirm pathological findings from current in vitro models or enable adaptations within these systems to obtain more accurate information on the effects of thermal stress on oocyte competence and embryo viability. In turn, this will facilitate a more effective implementation of both in vivo [331] and in vitro [332,333] therapies to mitigate the detrimental effects of heat stress on gamete and embryo biology.

### Technical Considerations in Bovine In Vitro Models of Heat Stress

In addition to using relevant temperatures that mimic in vivo heat stress in the reproductive tract and ovarian follicles, addressing technical factors is also crucial when developing in vitro models of heat stress. A relevant technical factor to consider in heat stress in vitro models is the abrupt shift to high temperatures that oocytes, sperm, and pre-implantation embryos endure in such experimental settings, which fails to accurately replicate the gradual circadian temperature increases experienced by cattle under heat stress conditions at the farm level. Few studies have explored the use of gradual temperature increases during experimental in vitro models of heat stress in cattle [143,157,194].

Finally, another important consideration in in vitro research is the pH elevation in culture media due to increased temperatures in systems designed to mimic cellular heat stress [76]. These pH alterations can adversely affect gamete and embryo physiology, potentially impairing successful IVEP outcomes [334,335,336,337,338]. Some research teams have addressed this issue by increasing CO₂ levels during in vitro culture, but the majority of studies do not implement this practice (Table 1 and Table 2) or monitor the pH values of the culture media during thermal stress [157].

## 8. Conclusions

The available evidence suggests that GV-stage oocytes are vulnerable to high temperatures, predominantly observed in *Bos taurus* raised in tropical or subtropical regions. However, there is a lack of studies addressing the potential detrimental effects of heat stress on primordial follicle viability. In vitro models examining the exposure of oocytes and pre-implantation embryos to heat stress have clearly demonstrated that elevated temperatures significantly diminish the capacity of both oocytes and early embryos to progress to the blastocyst stage. However, the accuracy of current in vitro bovine models in simulating follicular, uterine, and oviductal temperatures during heat stress is uncertain given the lack of in vivo data on these body compartments. As such, there is a possibility that the negative impact of heat stress on pre-implantation embryo development might not be due to a direct effect on the oocyte and embryo itself, but rather due to changes in the function of ovarian and uterine/oviductal tissues under thermal strain.

A clear cellular and molecular profile of blastocysts derived from in vitro heat-stressed oocytes and early embryos is difficult to establish due to the fact that variables of oocyte and embryo quality do not consistently respond to heat stress, together with conflicting gene expression patterns reported across in vitro studies. Future in vivo studies should employ protocols that accurately confirm whether individuals are experiencing heat stress, rather than relying solely on THI values. This approach is particularly pertinent given the presence of heat-tolerant cattle, even among high milk producers such as Holstein cattle.

## Figures and Tables

**Table 2:** In vitro bovine models analysing the effect of heat stress during the pre-implantation period on blastocyst production.

Temperature (°C)/CO_2_◆	Exposure Times (h)	Embryonic Stage Exposed to HS	Blastocyst Rate		Blastocyst Quality	Reference
•C: 39🛇 H: 41	12	2-cell	↓	C: 26–48% * H: 0–8% *	NR	[120]
		4–8-cell	↓	C: 24% * H: 10% *		
		CM	↔	C: 37% * H: 41% *		
•C: 38.5↺ H: 40, 41 H: ⮥39.5–40.5	3, 6, 9, 12	1-cell	↓↔ ^a^	C: 37% * H: 4–42%	NR	[194]
		2-cell	↓↔ ^a^	C: 33% * H: 1–38% *		
	⮥19	1-cell	↔	C: 33% * H: 32% *		
	⮥19 × 8 days	1-cell↱exB	↓	C: 33% * H: 17 *		
•C: 38.5🛇 H: 41	6	8–16-cell/EM	↓↑ ^b^	C: 42–58 H: 24–57	↔Cell number	[222]
•C: 38.5🛇 H: 41	9	16-cell/EM	↓	C: 19% * H: 10% *	NR	[225]
•C: 38.5🛇 H: 41	6	8–16-cell/EM	↓	C: 29–52% * H: 3–16% *	↓↔Cell number ^c^	[223]
•C: 38.5↺ H: 41	6	2-cell	↓	C: 50–59% * H: 7–19% *	NR	[221]
•C: 39🛇 H: ⮥39.5–41	⮥48	1-cell	↓	C: 12% H: 1%	NR	[226]
	⮥24	8–16-cell/EM	↓	C: 10% H: 4.5%		
•C: 38.5🛇 H: 41	6	8–16-cell/EM	↓	C: 25–31% * H: 6–17% *	↔Cell number	[227]
•C: 38.5🛇 H: 41	9	EM/CM	↓	C: 43 * H: 25 *	↓Cell number ↑Apoptosis	[228]
•C: 38.5↺ H: 41	9	2-cell	↓	C: 40–66% * H: 14–36% *	NR	[229]
•C: 38.5🛇 H: 41	6	1-cell	↓	C: 37% * H: 19% *	↑ROS ↓Cell number	[224]
		4–8-cell	↓	C: 37% * H: 23% *	↑ROS ↓Cell number	
		8–16-cell/EM	↔	C: 37% * H: 40%	↔ROS/Cell number	
		CM/EB	↔	C: 37% * H: 38% *	↔ROS/Cell number	
•C: 38.5🛇 H: 41	15	EM/CM	↓↔ ^d^	C: 60–67% * H: 42–56% *	NR	[230]
•C: 38.5🛇 H: 41.5	6	4–8-cell	↓	C: 37% H: 12%	↔Cell number	[231]
•C: 38.5↺ H: 41	9	2-cell	↓ ↔ ^e^	C: 24–70% * H: 0–81% *	NR	[232]
		EM/CM		C: 55–55% * H: 22–54% *:		
•C: 38.5🛇 H: 40.5	10	1-cell, 4-cell ^f^	↓	C: 10% H: 1%	NR	[233]
•C: 38.5🛇 H: 41, 42	15	2-cell	↓	C: 39% * H: 21% *	NR	[234]
		EM/CM	↓↔ ^g^	C: 57–87% * H: 47–48% *		
•C: 38.5🛇 H: 40	192	1-cell↱ExB	↓	C: 37% * H: 11% *	↔Cell number	[126]
	24	1-cell	↓	C: 30% * H: 16% *	↓Cell number	
		EM	↔	C: 29% * H: 26% *	↔Cell number	
•C: 38.5🛇 H: 40.5	10	8–16-cell/EM, EM/CM	↓↔ ^h^	C: 40–47% H: 17–31%	NR	[235]
•C: 38.5🛇 H: 40	8	EM	↓	C: 30% H: 28%	↔Cell number	[127]
•C: 38.5🛇 H: 41	6	8–16-cell/EM	↓ ↔ ^i^	C: 34–39% H: 25–33%	↔Cell number ↔Apoptosis	[236]
•C: 38.5🛇 H: 40.5	5	1-cell	↔	C: 26% H: 25%	NR	[219]
	10		↓	C: 24–26% H: 9–10%		
•C: 38.5↺ H: 40.5	12, 24	1-cell	↓	C: 27–29% * H: 18–22% *	NR	[220]
•C: 38.5🛇 H: 40.5	6	4–8-cell	↓	C: 37–41% H: 22–23%	↔Cell number ↑ROS/Apoptosis ↓Mitochondria activity	[237]
		ExB	↔	C: 41% H: 32%	↔Cell number ↑Apoptosis ↔ROS ↓Mitochondria activity	
•C: 38.5🛇 H: 40	20	1-cell	↓	C: 30–33 *% H: 10 *–11%	↔Cell number ↑Apoptosis	[198]
•C: 38.5🛇 H: 40.5	⮥6	1-cell	↓	C: 31% * H: 17% *	↔Cell number ↓IFNT expression ↑ROS	[157]
•C: 39🛇 H: 41	24	1–2-cell	↓	C: 34% H: 13%	Altered gene expression	[238]
•C: 38.5🛇 H: 41	15	1–2-cell	↓	C: 34% * H: 17% *	NR	[128]
		EM/CM	↔	C: 24% * H: 24% *		
•C: 38.3🛇 H: 39.8, 41.1	6	1-cell	↓↔ ^j^	C: 41% H: 30–39%	NR	[177]
	12		↓	C: 41% H: 7–37%		

C = control, HS = heat stress, NR = not reported, EM = early morula (M), CM = compact M, EB = early blastocyst, ExB = expanded blastocyst. ↓ = decrease compared to control, ↑ = increase compared to control, ↔ = no difference between groups. * Percentages are approximate, extracted from bar charts as numerical values were not provided in the paper. CO_2_**◆** indicates whether the study maintained 5% CO_2_ (🛇) or increased it (↺) during IVEC heat stress conditions. ⮥ = gradual increase in temperature was applied in the HS group rather than static high-temperature exposure. ↱ = from X embryonic stage to Y embryonic stage. ^a^ Study tested 3, 6, 9, and 12 h; heat stress impaired blastocyst production only at 9 and 12 h. ^b^ Blastocyst production decreased in Holstein but increased in Brahman oocytes. ^c^ Study compared Angus, Holstein, and Brahman, and cell number was affected only in Angus and Holstein. ^d^ C and HS groups contained dimethyl sulfoxide; HS had a negative effect on blastocyst production in just one experiment. ^e^ Comparing high and low oxygen (O_2_) levels, decreased blastocyst production was observed only at high O_2_ levels. ^f^ Embryos were heat stressed twice, first at 1-cell stage and then at 4-cell stage. ^g^ Only EM/CM were exposed to both 41 °C and 42 °C, and blastocyst production was decreased only at 42 °C. ^h^ The 8–16-cell/EM embryos were heat-stressed once or twice (2nd exposure at EM/CM stage); only double exposure affected blastocyst yield. ^i^ Study compared Nellore and Jersey; difference in blastocyst production was found only in Jersey cows. ^j^ During 6-h exposure, only 41.1 °C impaired blastocyst production.

## Data Availability

No new data were created or analysed in this study. Data sharing is not applicable to this article.

## References

[1] Rahimi J., Mutua J.Y., Notenbaert A.M.O., Dieng D., Butterbach-Bahl K. (2020). Will dairy cattle production in West Africa be challenged by heat stress in the future?. Clim. Change.

[2] Thornton P., Nelson G., Mayberry D., Herrero M. (2021). Increases in extreme heat stress in domesticated livestock species during the twenty-first century. Glob. Change Biol..

[3] Vargas Zeppetello L.R., Raftery A.E., Battisti D.S. (2022). Probabilistic projections of increased heat stress driven by climate change. Commun. Earth Environ..

[4] North M., Franke J., Ouweneel B., Trisos C. (2023). Global risk of heat stress to cattle from climate change. Environ. Res. Lett..

[5] Thornton P., Nelson G., Mayberry D., Herrero M. (2022). Impacts of heat stress on global cattle production during the 21st century: A modelling study. Lancet Planet. Health.

[6] Polsky L., von Keyserlingk M.A.G. (2017). Invited review: Effects of heat stress on dairy cattle welfare. J. Dairy Sci..

[7] dos Santos M.M., Souza-Junior J.B.F., Dantas M.R.T., de Macedo Costa L.L. (2021). An updated review on cattle thermoregulation: Physiological responses, biophysical mechanisms, and heat stress alleviation pathways. Environ. Sci. Pollut. Res..

[8] Shephard R., Maloney S. (2023). A review of thermal stress in cattle. Aust. Vet. J..

[9] Berman A., Folman Y., Kaim M., Mamen M., Herz Z., Wolfenson D., Arieli A., Graber Y. (1985). Upper Critical Temperatures and Forced Ventilation Effects for High-Yielding Dairy Cows in a Subtropical Climate. J. Dairy Sci..

[10] Igono M.O., Bjotvedt G., Sanford-Crane H.T. (1992). Environmental profile and critical temperature effects on milk production of Holstein cows in desert climate. Int. J. Biometeorol..

[11] Dikmen S., Hansen P.J. (2009). Is the temperature-humidity index the best indicator of heat stress in lactating dairy cows in a subtropical environment?. J. Dairy Sci..

[12] Sanderson M.G., Economou T., Salmon K.H., Jones S.E.O. (2017). Historical Trends and Variability in Heat Waves in the United Kingdom. Atmosphere.

[13] Wilcke R.A.I., Kjellström E., Lin C., Matei D., Moberg A., Tyrlis E. (2020). The extremely warm summer of 2018 in Sweden—Set in a historical context. Earth Syst. Dynam..

[14] Dunn R.J.H., Mead N.E., Willett K.M., Parker D.E. (2014). Analysis of heat stress in UK dairy cattle and impact on milk yields. Environ. Res. Lett..

[15] Kendon M. (2022). Unprecedented Extreme Heatwave, July 2022.

[16] Rowan T.N., Durbin H.J., Seabury C.M., Schnabel R.D., Decker J.E. (2021). Powerful detection of polygenic selection and evidence of environmental adaptation in US beef cattle. PLoS Genet..

[17] West J.W. (2003). Effects of Heat-Stress on Production in Dairy Cattle. J. Dairy Sci..

[18] Hahn G.L. (1999). Dynamic responses of cattle to thermal heat loads. J. Anim. Sci..

[19] Ahmed H., Tamminen L.-M., Emanuelson U. (2022). Temperature, productivity, and heat tolerance: Evidence from Swedish dairy production. Clim. Change.

[20] Lees A.M., Sejian V., Wallage A.L., Steel C.C., Mader T.L., Lees J.C., Gaughan J.B. (2019). The Impact of Heat Load on Cattle. Animals.

[21] Giannone C., Bovo M., Ceccarelli M., Torreggiani D., Tassinari P. (2023). Review of the Heat Stress-Induced Responses in Dairy Cattle. Animals.

[22] Dean L., Tarpoff A.J., Nickles K., Place S., Edwards-Callaway L. (2023). Heat Stress Mitigation Strategies in Feedyards: Use, Perceptions, and Experiences of Industry Stakeholders. Animals.

[23] Burhans W.S., Rossiter Burhans C.A., Baumgard L.H. (2022). Invited review: Lethal heat stress: The putative pathophysiology of a deadly disorder in dairy cattle. J. Dairy Sci..

[24] Bishop-Williams K.E., Berke O., Pearl D.L., Hand K., Kelton D.F. (2015). Heat stress related dairy cow mortality during heat waves and control periods in rural Southern Ontario from 2010–2012. BMC Vet. Res..

[25] Vitali A., Felici A., Esposito S., Bernabucci U., Bertocchi L., Maresca C., Nardone A., Lacetera N. (2015). The effect of heat waves on dairy cow mortality. J. Dairy Sci..

[26] Morignat E., Perrin J.B., Gay E., Vinard J.L., Calavas D., Hénaux V. (2014). Assessment of the impact of the 2003 and 2006 heat waves on cattle mortality in France. PLoS ONE.

[27] Crescio M.I., Forastiere F., Maurella C., Ingravalle F., Ru G. (2010). Heat-related mortality in dairy cattle: A case crossover study. Prev. Vet. Med..

[28] St-Pierre N.R., Cobanov B., Schnitkey G. (2003). Economic Losses from Heat Stress by US Livestock Industries1. J. Dairy Sci..

[29] Hristov A.N., Degaetano A.T., Rotz C.A., Hoberg E., Skinner R.H., Felix T., Li H., Patterson P.H., Roth G., Hall M. (2018). Climate change effects on livestock in the Northeast US and strategies for adaptation. Clim. Change.

[30] Mauger G., Bauman Y., Nennich T., Salathé E. (2015). Impacts of Climate Change on Milk Production in the United States. Prof. Geogr..

[31] Hempel S., Menz C., Pinto S., Galán E., Janke D., Estellés F., Müschner-Siemens T., Wang X., Heinicke J., Zhang G. (2019). Heat stress risk in European dairy cattle husbandry under different climate change scenarios—Uncertainties and potential impacts. Earth Syst. Dynam..

[32] De Rensis F., Saleri R., Garcia-Ispierto I., Scaramuzzi R., López-Gatius F. (2021). Effects of Heat Stress on Follicular Physiology in Dairy Cows. Animals.

[33] Dovolou E., Giannoulis T., Nanas I., Amiridis G.S. (2023). Heat Stress: A Serious Disruptor of the Reproductive Physiology of Dairy Cows. Animals.

[34] Roth Z. (2017). Effect of Heat Stress on Reproduction in Dairy Cows: Insights into the Cellular and Molecular Responses of the Oocyte. Annu. Rev. Anim. Biosci..

[35] Miętkiewska K., Kordowitzki P., Pareek C.S. (2022). Effects of Heat Stress on Bovine Oocytes and Early Embryonic Development—An Update. Cells.

[36] Boni R. (2019). Heat stress, a serious threat to reproductive function in animals and humans. Mol. Reprod. Dev..

[37] de Barros F.R.O., Paula-Lopes F.F. (2018). Cellular and epigenetic changes induced by heat stress in bovine preimplantation embryos. Mol. Reprod. Dev..

[38] Sakatani M. (2017). Effects of heat stress on bovine preimplantation embryos produced in vitro. J. Reprod. Dev..

[39] Naranjo-Gómez J.S., Uribe-García H.F., Herrera-Sánchez M.P., Lozano-Villegas K.J., Rodríguez-Hernández R., Rondón-Barragán I.S. (2021). Heat stress on cattle embryo: Gene regulation and adaptation. Heliyon.

[40] Fleming T.P., Watkins A.J., Velazquez M.A., Mathers J.C., Prentice A.M., Stephenson J., Barker M., Saffery R., Yajnik C.S., Eckert J.J. (2018). Origins of lifetime health around the time of conception: Causes and consequences. Lancet.

[41] Velazquez M.A., Fleming T.P., Watkins A.J. (2019). Periconceptional environment and the developmental origins of disease. J. Endocrinol..

[42] Rhoads M.L. (2020). Effects of periconceptional heat stress on primiparous and multiparous daughters of Holstein dairy cows. Theriogenology.

[43] Zou K., Yuan Z., Yang Z., Luo H., Sun K., Zhou L., Xiang J., Shi L., Yu Q., Zhang Y. (2009). Production of offspring from a germline stem cell line derived from neonatal ovaries. Nat. Cell Biol..

[44] White Y.A., Woods D.C., Takai Y., Ishihara O., Seki H., Tilly J.L. (2012). Oocyte formation by mitotically active germ cells purified from ovaries of reproductive-age women. Nat. Med..

[45] Yoshihara M., Wagner M., Damdimopoulos A., Zhao C., Petropoulos S., Katayama S., Kere J., Lanner F., Damdimopoulou P. (2023). The Continued Absence of Functional Germline Stem Cells in Adult Ovaries. Stem Cells.

[46] Hainaut M., Clarke H.J. (2021). Germ cells of the mammalian female: A limited or renewable resource?. Biol. Reprod..

[47] Mossa F., Evans A.C.O. (2023). Review: The ovarian follicular reserve—Implications for fertility in ruminants. Animal.

[48] Zhang T., He M., Zhang J., Tong Y., Chen T., Wang C., Pan W., Xiao Z. (2023). Mechanisms of primordial follicle activation and new pregnancy opportunity for premature ovarian failure patients. Front. Physiol..

[49] Juengel J.L., Smith P., Juengel J.L., Miyamoto A., Price C., Reynolds L.P., Smith M.F., Webb R. (2014). Formation of ovarian follicles in ruminants. Reproduction in Domestic Ruminants VIII.

[50] Rodgers R.J., Irving-Rodgers H.F. (2010). Morphological classification of bovine ovarian follicles. Reproduction.

[51] Costa C.B., Fair T., Seneda M.M. (2023). Review: Environment of the ovulatory follicle: Modifications and use of biotechnologies to enhance oocyte competence and increase fertility in cattle. Animal.

[52] Britt J.H. (2008). Oocyte development in cattle: Physiological and genetic aspects. Rev. Bras. De Zootec..

[53] Velazquez M.A. (2023). Nutritional Strategies to Promote Bovine Oocyte Quality for In Vitro Embryo Production: Do They Really Work?. Vet. Sci..

[54] Al-Katanani Y.M., Paula-Lopes F.F., Hansen P.J. (2002). Effect of Season and Exposure to Heat Stress on Oocyte Competence in Holstein Cows1. J. Dairy Sci..

[55] Rocha A., Randel R.D., Broussard J.R., Lim J.M., Blair R.M., Roussel J.D., Godke R.A., Hansel W. (1998). High environmental temperature and humidity decrease oocyte quality in Bos taurus but not in Bos taurus cows. Theriogenology.

[56] Gendelman M., Roth Z. (2012). In vivo vs. in vitro models for studying the effects of elevated temperature on the GV-stage oocyte, subsequent developmental competence and gene expression. Anim. Reprod. Sci..

[57] Gendelman M., Roth Z. (2012). Seasonal Effect on Germinal Vesicle-Stage Bovine Oocytes Is Further Expressed by Alterations in Transcript Levels in the Developing Embryos Associated with Reduced Developmental Competence1. Biol. Reprod..

[58] Gendelman M., Roth Z. (2012). Incorporation of Coenzyme Q10 into Bovine Oocytes Improves Mitochondrial Features and Alleviates the Effects of Summer Thermal Stress on Developmental Competence1. Biol. Reprod..

[59] Yaacobi-Artzi S., Kalo D., Roth Z. (2022). Seasonal variation in the morphokinetics of in-vitro-derived bovine embryos is associated with the blastocyst developmental competence and gene expression. Front. Reprod. Health.

[60] Pavani K., Carvalhais I., Faheem M., Chaveiro A., Reis F.V., da Silva F.M. (2015). Reproductive Performance of Holstein Dairy Cows Grazing in Dry-summer Subtropical Climatic Conditions: Effect of Heat Stress and Heat Shock on Meiotic Competence and In vitro Fertilization. Asian-Australas. J. Anim. Sci..

[61] Pavani K., Baron E., Correia P., Lourenço J., Bettencourt B.F., Sousa M., da Silva F. (2016). Gene expression, oocyte nuclear maturation and developmental competence of bovine oocytes and embryos produced after in vivo and in vitro heat shock. Zygote.

[62] Sa S.J., Jeong J., Cho J., Lee S.-H., Choi I. (2018). Heat waves impair cytoplasmic maturation of oocytes and preimplantation development in Korean native cattle (Hanwoo). Korean J. Agric. Sci..

[63] Elgendy O., Kitahara G., Taniguchi S., Osawa T. (2022). 5-Aminolevulinic acid combined with sodium ferrous citrate mitigates effects of heat stress on bovine oocyte developmental competence. J. Reprod. Dev..

[64] Báez F., López Darriulat R., Rodríguez-Osorio N., Viñoles C. (2022). Effect of season on germinal vesicle stage, quality, and subsequent in vitro developmental competence in bovine cumulus-oocyte complexes. J. Therm. Biol..

[65] Zeron Y., Ocheretny A., Kedar O., Borochov A., Sklan D., Arav A. (2001). Seasonal changes in bovine fertility: Relation to developmental competence of oocytes, membrane properties and fatty acid composition of follicles. Reproduction.

[66] Ferreira R.M., Ayres H., Chiaratti M.R., Ferraz M.L., Araújo A.B., Rodrigues C.A., Watanabe Y.F., Vireque A.A., Joaquim D.C., Smith L.C. (2011). The low fertility of repeat-breeder cows during summer heat stress is related to a low oocyte competence to develop into blastocysts. J. Dairy Sci..

[67] Berling F., Castro F.C.d., Oliveira A.C.d.S. (2022). Infuence of heat stress on in vitro oocyte and embryo production in high-yielding Holstein cows. Ciência Anim. Bras..

[68] Morales-Cruz J.L., Calderon-Leyva G., Angel-García O., Guillen-Muñoz J.M., Santos-Jimenez Z., Mellado M., Pessoa L.G., Guerrero-Gallego H.Z. (2023). The Effect of Month of Harvesting and Temperature–Humidity Index on the Number and Quality of Oocytes and In Vitro Embryo Production in Holstein Cows and Heifers. Biology.

[69] Guerrero-Gallego H.Z., Calderón-Leyva G., Ángel-García O., Guillen-Muñoz J., Leyva C., Mellado M., Pedroso R., Pessoa L.G., Esparza C., Morales J. (2021). Effect of Season on Quantity and Competence of Oocytes Recovered Transvaginally from Holstein Cows for In vitro Fertilization. Indian J. Anim. Res..

[70] Gendelman M., Aroyo A., Yavin S., Roth Z. (2010). Seasonal effects on gene expression, cleavage timing, and developmental competence of bovine preimplantation embryos. Reproduction.

[71] Ferreira R.M., Chiaratti M.R., Macabelli C.H., Rodrigues C.A., Ferraz M.L., Watanabe Y.F., Smith L.C., Meirelles F.V., Baruselli P.S. (2016). The Infertility of Repeat-Breeder Cows During Summer Is Associated with Decreased Mitochondrial DNA and Increased Expression of Mitochondrial and Apoptotic Genes in Oocytes1. Biol. Reprod..

[72] Diaz F.A., Gutierrez-Castillo E.J., Foster B.A., Hardin P.T., Bondioli K.R., Jiang Z. (2021). Evaluation of Seasonal Heat Stress on Transcriptomic Profiles and Global DNA Methylation of Bovine Oocytes. Front. Genet..

[73] Torres-Júnior J.R.d.S., Pires M.d.F.A., de Sá W.F., Ferreira A.d.M., Viana J.H.M., Camargo L.S.A., Ramos A.A., Folhadella I.M., Polisseni J., de Freitas C. (2008). Effect of maternal heat-stress on follicular growth and oocyte competence in Bos indicus cattle. Theriogenology.

[74] Payton R.R., Romar R., Coy P., Saxton A.M., Lawrence J.L., Edwards J.L. (2004). Susceptibility of Bovine Germinal Vesicle-Stage Oocytes from Antral Follicles to Direct Effects of Heat Stress In Vitro1. Biol. Reprod..

[75] Lima R.S., Risolia P.H.B., Ispada J., Assumpção M., Visintin J.A., Orlandi C., Paula-Lopes F.F. (2017). Role of insulin-like growth factor 1 on cross-bred Bos indicus cattle germinal vesicle oocytes exposed to heat shock. Reprod. Fertil. Dev..

[76] Kawano K., Sakaguchi K., Madalitso C., Ninpetch N., Kobayashi S., Furukawa E., Yanagawa Y., Katagiri S. (2022). Effect of heat exposure on the growth and developmental competence of bovine oocytes derived from early antral follicles. Sci. Rep..

[77] Paes V.M., Vieira L.A., Correia H.H.V., Sa N.A.R., Moura A.A.A., Sales A.D., Rodrigues A.P.R., Magalhães-Padilha D.M., Santos F.W., Apgar G.A. (2016). Effect of heat stress on the survival and development of in vitro cultured bovine preantral follicles and on in vitro maturation of cumulus–oocyte complex. Theriogenology.

[78] Aguiar L.H.d., Hyde K.A., Pedroza G.H., Denicol A.C. (2020). Heat stress impairs in vitro development of preantral follicles of cattle. Anim. Reprod. Sci..

[79] Cardone D.A., Cáceres A.R.R., Sanhueza M.A., Bruna F.A., Laconi M.R. (2022). Effects of short-term in vitro heat stress on bovine preantral follicles. Livest. Sci..

[80] Tang L., Bai X., Xie X., Chen G., Jia X., Lei M., Li C., Lai S. (2022). Negative effects of heat stress on ovarian tissue in female rabbit. Front. Vet. Sci..

[81] Wolfenson D., Roth Z. (2019). Impact of heat stress on cow reproduction and fertility. Anim. Front..

[82] Khan A., Dou J., Wang Y., Jiang X., Khan M.Z., Luo H., Usman T., Zhu H. (2020). Evaluation of heat stress effects on cellular and transcriptional adaptation of bovine granulosa cells. J. Anim. Sci. Biotechnol..

[83] Khan A., Khan M.Z., Dou J., Umer S., Xu H., Sammad A., Zhu H.B., Wang Y. (2020). RNAi-Mediated Silencing of Catalase Gene Promotes Apoptosis and Impairs Proliferation of Bovine Granulosa Cells under Heat Stress. Animals.

[84] Sammad A., Luo H., Hu L., Zhao S., Gong J., Umer S., Khan A., Zhu H., Wang Y. (2022). Joint Transcriptome and Metabolome Analysis Prevails the Biological Mechanisms Underlying the Pro-Survival Fight in In Vitro Heat-Stressed Granulosa Cells. Biology.

[85] Sammad A., Luo H., Hu L., Zhu H., Wang Y. (2022). Transcriptome Reveals Granulosa Cells Coping through Redox, Inflammatory and Metabolic Mechanisms under Acute Heat Stress. Cells.

[86] Stamperna K., Giannoulis T., Cañon-Beltrán K., Dovolou E., Kalemkeridou M., Nanas I., Rizos D., Moutou K.A., Mamuris Z., Amiridis G.S. (2022). Oviductal epithelial cells transcriptome and extracellular vesicles characterization during thermoneutral and heat stress conditions in dairy cows. Theriogenology.

[87] Ealy A.D., Drost M., Hansen P.J. (1993). Developmental changes in embryonic resistance to adverse effects of maternal heat stress in cows. J. Dairy Sci..

[88] Leipold H.W., Dennis S.M., Huston K., Dayton A.D. (1974). Hereditary Bovine Syndactyly. II. Hyperthermia. J. Dairy Sci..

[89] Roth Z., Hansen P.J. (2004). Sphingosine 1-phosphate protects bovine oocytes from heat shock during maturation. Biol. Reprod..

[90] Swain J.E., Pool T.B. (2008). ART failure: Oocyte contributions to unsuccessful fertilization. Hum. Reprod. Update.

[91] He M., Zhang T., Yang Y., Wang C. (2021). Mechanisms of Oocyte Maturation and Related Epigenetic Regulation. Front. Cell Dev. Biol..

[92] Ferreira E.M., Vireque A.A., Adona P.R., Meirelles F.V., Ferriani R.A., Navarro P.A.A.S. (2009). Cytoplasmic maturation of bovine oocytes: Structural and biochemical modifications and acquisition of developmental competence. Theriogenology.

[93] Stroebech L., Mazzoni G., Pedersen H., Freude K., Kadarmideen H., Callesen H., Hyttel P. (2015). In vitro production of bovine embryos: Revisiting oocyte development and application of systems biology. Anim. Reprod..

[94] Lonergan P., Fair T. (2016). Maturation of Oocytes in Vitro. Annu. Rev. Anim. Biosci..

[95] Dutt R.H. (1964). Detrimental effects of high ambient temperature on fertility and early embryo survival in sheep. Int. J. Biometeorol..

[96] Dutt R.H. (1963). Critical Period for Early Embryo Mortality in Ewes Exposed to High Ambient Temperature. J. Anim. Sci..

[97] Putney D.J., Mullins S., Thatcher W.W., Drost M., Gross T.S. (1989). Embryonic development in superovulated dairy cattle exposed to elevated ambient temperatures between the onset of estrus and insemination. Anim. Reprod. Sci..

[98] Nabenishi H., Ohta H., Nishimoto T., Morita T., Ashizawa K., Tsuzuki Y. (2011). Effect of the temperature-humidity index on body temperature and conception rate of lactating dairy cows in southwestern Japan. J. Reprod. Dev..

[99] Wise M.E., Armstrong D.V., Huber J.T., Hunter R., Wiersma F. (1988). Hormonal alterations in the lactating dairy cow in response to thermal stress. J. Dairy Sci..

[100] Gilad E., Meidan R., Berman A., Graber Y., Wolfenson D. (1993). Effect of heat stress on tonic and GnRH-induced gonadotrophin secretion in relation to concentration of oestradiol in plasma of cyclic cows. J. Reprod. Fertil..

[101] Honig H., Ofer L., Kaim M., Jacobi S., Shinder D., Gershon E. (2016). The effect of cooling management on blood flow to the dominant follicle and estrous cycle length at heat stress. Theriogenology.

[102] Jitjumnong J., Moonmanee T., Sudwan P., Mektrirat R., Osathanunkul M., Navanukraw C., Panatuk J., Yama P., Pirokad W., Warittha U.K. (2020). Associations among thermal biology, preovulatory follicle diameter, follicular and luteal vascularities, and sex steroid hormone concentrations during preovulatory and postovulatory periods in tropical beef cows. Anim. Reprod. Sci..

[103] Darbaz I., Sayiner S., Ergene O., Seyrek Intas K., Zabitler F., Evci E.C., Aslan S. (2021). The Effect of Comfort- and Hot-Period on the Blood Flow of Corpus Luteum (CL) in Cows Treated by an OvSynch Protocol. Animals.

[104] Badinga L., Thatcher W.W., Diaz T., Drost M., Wolfenson D. (1993). Effect of environmental heat stress on follicular development and steroidogenesis in lactating Holstein cows. Theriogenology.

[105] Wilson S.J., Kirby C.J., Koenigsfeld A.T., Keisler D.H., Lucy M.C. (1998). Effects of controlled heat stress on ovarian function of dairy cattle. 2. Heifers. J. Dairy Sci..

[106] Guzeloglu A., Ambrose J.D., Kassa T., Diaz T., Thatcher M.J., Thatcher W.W. (2001). Long-term follicular dynamics and biochemical characteristics of dominant follicles in dairy cows subjected to acute heat stress. Anim. Reprod. Sci..

[107] Shehab-El-Deen M.A.M.M., Leroy J.L.M.R., Fadel M.S., Saleh S.Y.A., Maes D., Van Soom A. (2010). Biochemical changes in the follicular fluid of the dominant follicle of high producing dairy cows exposed to heat stress early post-partum. Anim. Reprod. Sci..

[108] Wilson S.J., Marion R.S., Spain J.N., Spiers D.E., Keisler D.H., Lucy M.C. (1998). Effects of controlled heat stress on ovarian function of dairy cattle. 1. Lactating cows. J. Dairy Sci..

[109] Roth Z., Meidan R., Braw-Tal R., Wolfenson D. (2000). Immediate and delayed effects of heat stress on follicular development and its association with plasma FSH and inhibin concentration in cows. J. Reprod. Fertil..

[110] Wolfenson D., Thatcher W.W., Badinga L., Savi0 J.D., Meidan R., Lew B.J., Braw-tal R., Berman A. (1995). Effect of Heat Stress on Follicular Development during the Estrous Cycle in Lactating Dairy Cattle1. Biol. Reprod..

[111] Wolfenson D., Lew B.J., Thatcher W.W., Graber Y., Meidan R. (1997). Seasonal and acute heat stress effects on steroid production by dominant follicles in cows. Anim. Reprod. Sci..

[112] Roth Z., Meidan R., Shaham-Albalancy A., Braw-Tal R., Wolfenson D. (2001). Delayed effect of heat stress on steroid production in medium-sized and preovulatory bovine follicles. Reproduction.

[113] Bridges P.J., Brusie M.A., Fortune J.E. (2005). Elevated temperature (heat stress) in vitro reduces androstenedione and estradiol and increases progesterone secretion by follicular cells from bovine dominant follicles. Domest. Anim. Endocrinol..

[114] Rispoli L.A., Edwards J.L., Pohler K.G., Russell S., Somiari R.I., Payton R.R., Schrick F.N. (2019). Heat-induced hyperthermia impacts the follicular fluid proteome of the periovulatory follicle in lactating dairy cows. PLoS ONE.

[115] de Castro E.P.L.A., Andrzejewski J., Julian D., Spicer L.J., Hansen P.J. (2008). Oxygen and steroid concentrations in preovulatory follicles of lactating dairy cows exposed to acute heat stress. Theriogenology.

[116] Trout J.P., McDowell L.R., Hansen P.J. (1998). Characteristics of the estrous cycle and antioxidant status of lactating Holstein cows exposed to heat stress. J. Dairy Sci..

[117] Mogollón H.D.G., Ferrazza R.A., Vallejo V.H., Destro F.C., Ochoa J.C., Nogueira C., Carvalho R.F., Moraes L.N., Rizzoto G., Sartori R. (2020). Short communication: Heat stress does not affect induced luteolysis in Holstein cows. J. Dairy Sci..

[118] Alves M.F., Fernandes Gonçalves R., Labadessa Pavão D., Gimenes Palazzi E., Souza F., Ribeiro de Queiróz R.K., D’ Angelo M., de Achilles M.A. (2013). Effect of heat stress on the maturation, fertilization and development rates of in vitro produced bovine embryos. Open J. Anim. Sci..

[119] Cebrian-Serrano A., Salvador I., Raga E., Dinnyes A., Silvestre M.A. (2013). Beneficial effect of melatonin on blastocyst in vitro production from heat-stressed bovine oocytes. Reprod. Domest. Anim..

[120] Edwards J.L., Hansen P.J. (1997). Differential responses of bovine oocytes and preimplantation embryos to heat shock. Mol. Reprod. Dev..

[121] Edwards J.L., Hansen P.J. (1996). Elevated Temperature Increases Heat Shock Protein 70 Synthesis in Bovine Two-Cell Embryos and Compromises Function of Maturing Oocytes. Biol. Reprod..

[122] Báez F., Camargo Á., Reyes A.L., Márquez A., Paula-Lopes F., Viñoles C. (2019). Time-dependent effects of heat shock on the zona pellucida ultrastructure and in vitro developmental competence of bovine oocytes. Reprod. Biol..

[123] Edwards J.L., Bogart A.N., Rispoli L.A., Saxton A.M., Schrick F.N. (2009). Developmental competence of bovine embryos from heat-stressed ova. J. Dairy Sci..

[124] Yaacobi-Artzi S., Shimoni C., Kalo D., Hansen P.J., Roth Z. (2020). Melatonin slightly alleviates the effect of heat shock on bovine oocytes and resulting blastocysts. Theriogenology.

[125] Baruselli P.S., Ferreira R.M., Vieira L.M., Souza A.H., Bó G.A., Rodrigues C.A. (2020). Use of embryo transfer to alleviate infertility caused by heat stress. Theriogenology.

[126] Sakatani M., Alvarez N.V., Takahashi M., Hansen P.J. (2012). Consequences of physiological heat shock beginning at the zygote stage on embryonic development and expression of stress response genes in cattle. J. Dairy Sci..

[127] Sakatani M., Bonilla L., Dobbs K.B., Block J., Ozawa M., Shanker S., Yao J., Hansen P.J. (2013). Changes in the transcriptome of morula-stage bovine embryos caused by heat shock: Relationship to developmental acquisition of thermotolerance. Reprod. Biol. Endocrinol..

[128] Sosa F., Hansen P.J. (2023). Colony stimulating factor 2 protects the preimplantation bovine embryo from heat shock. Zygote.

[129] Edwards J.L., Saxton A.M., Lawrence J.L., Payton R.R., Dunlap J.R. (2005). Exposure to a Physiologically Relevant Elevated Temperature Hastens In Vitro Maturation in Bovine Oocytes. J. Dairy Sci..

[130] Pöhland R., Souza-Cácares M.B., Datta T.K., Vanselow J., Martins M.I.M., Silva W.A.L.d., Cardoso C.J.T., Melo-Sterza F.d.A. (2020). Influence of long-term thermal stress on the in vitro maturation on embryo development and Heat Shock Protein abundance in zebu cattle. Anim. Reprod..

[131] Marble N., Marle-Köster V., Kgomotso L., Lotus M.M., Christina L.K., Mpho P.C., Lucky N.T. (2022). The impact of high temperature on the development and quality of in vitro matured beef cattle oocytes and their potential to subsequent embryonic development. Adv. Anim. Vet. Sci..

[132] Lee J., Kim D., Son J., Kim D., Jeon E., Jung D., Han M., Ha S., Hwang S., Choi I. (2023). Effects of heat stress on conception in Holstein and Jersey cattle and oocyte maturation in vitro. J. Anim. Sci. Technol..

[133] Rienzi L., Ubaldi F., Iacobelli M., Minasi M.G., Romano S., Greco E. (2005). Meiotic spindle visualization in living human oocytes. Reprod. Biomed. Online.

[134] Lawrence J.L., Payton R.R., Godkin J.D., Saxton A.M., Schrick F.N., Edwards J.L. (2004). Retinol Improves Development of Bovine Oocytes Compromised by Heat Stress During Maturation. J. Dairy Sci..

[135] Roth Z., Hansen P.J. (2004). Involvement of apoptosis in disruption of developmental competence of bovine oocytes by heat shock during maturation. Biol. Reprod..

[136] Roth Z., Hansen P.J. (2005). Disruption of nuclear maturation and rearrangement of cytoskeletal elements in bovine oocytes exposed to heat shock during maturation. Reproduction.

[137] Schrock G.E., Saxton A.M., Schrick F.N., Edwards J.L. (2007). Early in vitro fertilization improves development of bovine ova heat stressed during in vitro maturation. J. Dairy Sci..

[138] Soto P., Smith L.C. (2009). BH4 peptide derived from Bcl-xL and Bax-inhibitor peptide suppresses apoptotic mitochondrial changes in heat stressed bovine oocytes. Mol. Reprod. Dev..

[139] Zhandi M., Towhidi A., Nasr-Esfahani M.H., Eftekhari-Yazdi P., Zare-Shahneh A. (2009). Unexpected detrimental effect of Insulin like growth factor-1 on bovine oocyte developmental competence under heat stress. J. Assist. Reprod. Genet..

[140] Kalo D., Roth Z. (2011). Involvement of the sphingolipid ceramide in heat-shock-induced apoptosis of bovine oocytes. Reprod. Fertil. Dev..

[141] Payton R.R., Rispoli L.A., Saxton A.M., Edwards J.L. (2011). Impact of heat stress exposure during meiotic maturation on oocyte, surrounding cumulus cell, and embryo RNA populations. J. Reprod. Dev..

[142] Rispoli L.A., Lawrence J.L., Payton R.R., Saxton A.M., Schrock G.E., Schrick F.N., Middlebrooks B.W., Dunlap J.R., Parrish J.J., Edwards J.L. (2011). Disparate consequences of heat stress exposure during meiotic maturation: Embryo development after chemical activation vs fertilization of bovine oocytes. Reproduction.

[143] Nabenishi H., Ohta H., Nishimoto T., Morita T., Ashizawa K., Tsuzuki Y. (2012). The effects of cysteine addition during in vitro maturation on the developmental competence, ROS, GSH and apoptosis level of bovine oocytes exposed to heat stress. Zygote.

[144] Balboula A.Z., Yamanaka K., Sakatani M., Kawahara M., Hegab A.O., Zaabel S.M., Takahashi M. (2013). Cathepsin B activity has a crucial role in the developmental competence of bovine cumulus-oocyte complexes exposed to heat shock during in vitro maturation. Reproduction.

[145] Meiyu Q., Liu D., Roth Z. (2015). IGF-I slightly improves nuclear maturation and cleavage rate of bovine oocytes exposed to acute heat shock in vitro. Zygote.

[146] Rodrigues T.A., Ispada J., Risolia P.H.B., Rodrigues M.T., Lima R.S., Assumpção M.E.O.A., Visintin J.A., Paula-Lopes F.F. (2016). Thermoprotective effect of insulin-like growth factor 1 on in vitro matured bovine oocyte exposed to heat shock. Theriogenology.

[147] Ascari I.J., Alves N.G., Jasmin J., Lima R.R., Quintão C.C.R., Oberlender G., Moraes E.A., Camargo L.S.A. (2017). Addition of insulin-like growth factor I to the maturation medium of bovine oocytes subjected to heat shock: Effects on the production of reactive oxygen species, mitochondrial activity and oocyte competence. Domest. Anim. Endocrinol..

[148] Kim M.-S., Kim C.-L., Seong H.-H., Kim N., Kim S.W. (2017). Effects of Heat Stress on the Developmental Competence of Bovine Cumulus-Oocyte Complex During in vitro Maturation. J. Embryo Transf..

[149] Vendrell-Flotats M., Arcarons N., Barau E., López-Béjar M., Mogas T. (2017). Effect of heat stress during in vitro maturation on developmental competence of vitrified bovine oocytes. Reprod. Domest. Anim..

[150] Ispada J., Rodrigues T.A., Risolia P.H.B., Lima R.S., Gonçalves D.R., Rettori D., Nichi M., Feitosa W.B., Paula-Lopes F.F. (2018). Astaxanthin counteracts the effects of heat shock on the maturation of bovine oocytes. Reprod. Fertil. Dev..

[151] Payton R.R., Rispoli L.A., Nagle K.A., Gondro C., Saxton A.M., Voy B.H., Edwards J.L. (2018). Mitochondrial-related consequences of heat stress exposure during bovine oocyte maturation persist in early embryo development. J. Reprod. Dev..

[152] Camargo L.S.A., Aguirre-Lavin T., Adenot P., Araujo T.D., Mendes V.R.A., Louro I.D., Beaujean N., Souza E.D. (2019). Heat shock during in vitro maturation induces chromatin modifications in the bovine embryo. Reproduction.

[153] Camargo L.S.A., Costa F.Q., Munk M., Wohlres-Viana S., Serapião R.V., Carvalho B.C., Campos Jr P.H., Vieira A.C., Nogueira L.A.G., Viana J.H.M. (2019). Contrasting effects of heat shock during in vitro maturation on development of in vitro-fertilized and parthenogenetic bovine embryos. Reprod. Domest. Anim..

[154] Cavallari F.C., Leal C.L.V., Zvi R., Hansen P.J. (2019). Effects of melatonin on production of reactive oxygen species and developmental competence of bovine oocytes exposed to heat shock and oxidative stress during in vitro maturation. Zygote.

[155] Rodrigues T.A., Tuna K.M., Alli A.A., Tribulo P., Hansen P.J., Koh J., Paula-Lopes F.F. (2019). Follicular fluid exosomes act on the bovine oocyte to improve oocyte competence to support development and survival to heat shock. Reprod. Fertil. Dev..

[156] Abazarikia A.H., Zhandi M., Shakeri M., Towhidi A., Yousefi A.R. (2020). In vitro supplementation of trans-10, cis-12 conjugated linoleic acid ameliorated deleterious effect of heat stress on bovine oocyte developmental competence. Theriogenology.

[157] Amaral C.S., Koch J., Correa Júnior E.E., Bertolin K., Mujica L.K.S., Fiorenza M.F., Rosa S.G., Nogueira C.W., Comim F.V., Portela V.V.M. (2020). Heat stress on oocyte or zygote compromises embryo development, impairs interferon tau production and increases reactive oxygen species and oxidative stress in bovine embryos produced in vitro. Mol. Reprod. Dev..

[158] Latorraca L.B., Feitosa W.B., Mariano C., Moura M.T., Fontes P.K., Nogueira M.F.G., Paula-Lopes F.F. (2020). Autophagy is a pro-survival adaptive response to heat shock in bovine cumulus-oocyte complexes. Sci. Rep..

[159] Stamperna K., Giannoulis T., Nanas I., Kalemkeridou M., Dadouli K., Moutou K., Amiridis G.S., Dovolou E. (2020). Short term temperature elevation during IVM affects embryo yield and alters gene expression pattern in oocytes, cumulus cells and blastocysts in cattle. Theriogenology.

[160] Abazarikia A., Zhandi M., Towhidi A., Shakeri M., Yousefi A.R., Aliyan A. (2021). Conjugated linoleic acid improves meiotic spindle morphology and developmental competence of heat-stressed bovine oocyte. Theriogenology.

[161] Novaes M.A.S., Lima L.F., Sá N.A.R., Ferreira A.C.A., Paes V.M., Souza J.F., Alves B.G., Gramosa N.V., Torres C.A.A., Pukazhenthi B. (2021). Impact of ethanol and heat stress-dependent effect of ultra-diluted Arnica montana 6 cH on in vitro embryo production in cattle. Theriogenology.

[162] Rowinski J.R., Rispoli L.A., Payton R.R., Schneider L.G., Schrick F.N., McLean K.J., Edwards J.L. (2021). Impact of an acute heat shock during in vitro maturation on interleukin 6 and its associated receptor component transcripts in bovine cumulus-oocyte complexes. Anim. Reprod..

[163] Stamperna K., Dovolou E., Giannoulis T., Kalemkeridou M., Nanas I., Dadouli K., Moutou K., Mamuris Z., Amiridis G.S. (2021). Developmental competence of heat stressed oocytes from Holstein and Limousine cows matured in vitro. Reprod. Domest. Anim..

[164] Stamperna K., Giannoulis T., Dovolou E., Kalemkeridou M., Nanas I., Dadouli K., Moutou K., Mamuris Z., Amiridis G.S. (2021). Heat Shock Protein 70 Improves In Vitro Embryo Yield and Quality from Heat Stressed Bovine Oocytes. Animals.

[165] Feng X., Li C., Zhang H., Zhang P., Shahzad M., Du W., Zhao X. (2024). Heat-Stress Impacts on Developing Bovine Oocytes: Unraveling Epigenetic Changes, Oxidative Stress, and Developmental Resilience. Int. J. Mol. Sci..

[166] Mzedawee H.R.H., Kowsar R., Moradi-Hajidavaloo R., Shiasi-Sardoabi R., Sadeghi K., Nasr-Esfahani M.H., Hajian M. (2024). Heat shock interferes with the amino acid metabolism of bovine cumulus-oocyte complexes in vitro: A multistep analysis. Amino Acids.

[167] Ahmed J.A., Dutta D., Nashiruddullah N. (2016). Comparative efficacy of antioxidant retinol, melatonin, and zinc during in vitro maturation of bovine oocytes under induced heat stress. Turk. J. Vet. Anim. Sci..

[168] Maya-Soriano M.J., Taberner E., López-Béjar M. (2013). Retinol improves in vitro oocyte nuclear maturation under heat stress in heifers. Zygote.

[169] Andreu-Vázquez C., López-Gatius F., García-Ispierto I., Maya-Soriano M.J., Hunter R.H.F., López-Béjar M. (2010). Does heat stress provoke the loss of a continuous layer of cortical granules beneath the plasma membrane during oocyte maturation?. Zygote.

[170] Hooper L.M., Payton R.R., Rispoli L.A., Saxton A.M., Edwards J.L. (2015). Impact of heat stress on germinal vesicle breakdown and lipolytic changes during in vitro maturation of bovine oocytes. J. Reprod. Dev..

[171] Turathum B., Gao E.M., Chian R.C. (2021). The Function of Cumulus Cells in Oocyte Growth and Maturation and in Subsequent Ovulation and Fertilization. Cells.

[172] Martinez C.A., Rizos D., Rodriguez-Martinez H., Funahashi H. (2023). Oocyte-cumulus cells crosstalk: New comparative insights. Theriogenology.

[173] Vanselow J., Vernunft A., Koczan D., Spitschak M., Kuhla B. (2016). Exposure of Lactating Dairy Cows to Acute Pre-Ovulatory Heat Stress Affects Granulosa Cell-Specific Gene Expression Profiles in Dominant Follicles. PLoS ONE.

[174] Klabnik J.L., Christenson L.K., Gunewardena S.S.A., Pohler K.G., Rispoli L.A., Payton R.R., Moorey S.E., Neal Schrick F., Edwards J.L. (2022). Heat-induced increases in body temperature in lactating dairy cows: Impact on the cumulus and granulosa cell transcriptome of the periovulatory follicle. J. Anim. Sci..

[175] Campen K.A., Abbott C.R., Rispoli L.A., Payton R.R., Saxton A.M., Edwards J.L. (2018). Heat stress impairs gap junction communication and cumulus function of bovine oocytes. J. Reprod. Dev..

[176] Rispoli L.A., Payton R.R., Gondro C., Saxton A.M., Nagle K.A., Jenkins B.W., Schrick F.N., Edwards J.L. (2013). Heat stress effects on the cumulus cells surrounding the bovine oocyte during maturation: Altered matrix metallopeptidase 9 and progesterone production. Reproduction.

[177] Wrzecińska M., Kowalczyk A., Kordan W., Cwynar P., Czerniawska-Piątkowska E. (2023). Disorder of Biological Quality and Autophagy Process in Bovine Oocytes Exposed to Heat Stress and the Effectiveness of In Vitro Fertilization. Int. J. Mol. Sci..

[178] Suzuki H., Ju J.-C., Parks J.E., Yang X. (1998). Surface Ultrastructural Characteristics of Bovine Oocytes Following Heat Shock. J. Reprod. Dev..

[179] Ju J.C., Jiang S., Tseng J.K., Parks J.E., Yang X. (2005). Heat shock reduces developmental competence and alters spindle configuration of bovine oocytes. Theriogenology.

[180] Tseng J.K., Chen C.H., Chou P.C., Yeh S.P., Ju J.C. (2004). Influences of follicular size on parthenogenetic activation and in vitro heat shock on the cytoskeleton in cattle oocytes. Reprod. Domest. Anim..

[181] Held-Hoelker E., Ghanem N., Haake L., Salilew-Wondim D., Kurzella J., Tholen E., Große-Brinkhaus C., Rings F., Hoelker M. (2024). Sustainable effect of heat stress during maturation on the bioenergetics profile of bovine blastocysts. Reprod. Fertil. Dev..

[182] Ju J.C., Parks J.E., Yang X. (1999). Thermotolerance of IVM-derived bovine oocytes and embryos after short-term heat shock. Mol. Reprod. Dev..

[183] Yuan S., Wang Z., Peng H., Ward S.M., Hennig G.W., Zheng H., Yan W. (2021). Oviductal motile cilia are essential for oocyte pickup but dispensable for sperm and embryo transport. Proc. Natl. Acad. Sci. USA.

[184] Hawk H.W. (1987). Transport and fate of spermatozoa after insemination of cattle. J. Dairy Sci..

[185] Pérez-Cerezales S., Ramos-Ibeas P., Acuña O.S., Avilés M., Coy P., Rizos D., Gutiérrez-Adán A. (2018). The oviduct: From sperm selection to the epigenetic landscape of the embryo. Biol. Reprod..

[186] Vallet-Buisan M., Mecca R., Jones C., Coward K., Yeste M. (2023). Contribution of semen to early embryo development: Fertilization and beyond. Hum. Reprod. Update.

[187] Siu K.K., Serrão V.H.B., Ziyyat A., Lee J.E. (2021). The cell biology of fertilization: Gamete attachment and fusion. J. Cell Biol..

[188] Trebichalská Z., Holubcová Z. (2020). Perfect date—The review of current research into molecular bases of mammalian fertilization. J. Assist. Reprod. Genet..

[189] Deneke V.E., Pauli A. (2021). The Fertilization Enigma: How Sperm and Egg Fuse. Annu. Rev. Cell Dev. Biol..

[190] Evans J.P. (2020). Preventing polyspermy in mammalian eggs—Contributions of the membrane block and other mechanisms. Mol. Reprod. Dev..

[191] Bianchi E., Wright G.J. (2016). Sperm Meets Egg: The Genetics of Mammalian Fertilization. Annu. Rev. Genet..

[192] Clift D., Schuh M. (2013). Restarting life: Fertilization and the transition from meiosis to mitosis. Nat. Rev. Mol. Cell Biol..

[193] Moghaddam A., Karimi I., Pooyanmehr M. (2009). Effects of short-term cooling on pregnancy rate of dairy heifers under summer heat stress. Vet. Res. Commun..

[194] Rivera R.M., Hansen P.J. (2001). Development of cultured bovine embryos after exposure to high temperatures in the physiological range. Reproduction.

[195] Sugiyama S., McGowan M., Phillips N., Kafi M., Young M. (2007). Effects of increased ambient temperature during IVM and/or IVF on the in vitro development of bovine zygotes. Reprod. Domest. Anim..

[196] Lenz R.W., Ball G.D., Leibfried M.L., Ax R.L., First N.L. (1983). In Vitro Maturation and Fertilization of Bovine Oocytes are Temperature-Dependent Processes1. Biol. Reprod..

[197] Sakatani M., Yamanaka K., Balboula A.Z., Takenouchi N., Takahashi M. (2015). Heat stress during in vitro fertilization decreases fertilization success by disrupting anti-polyspermy systems of the oocytes. Mol. Reprod. Dev..

[198] Yamanaka K.I., Khatun H., Egashira J., Balboula A.Z., Tatemoto H., Sakatani M., Takenouchi N., Wada Y., Takahashi M. (2018). Heat-shock-induced cathepsin B activity during IVF and culture compromises the developmental competence of bovine embryos. Theriogenology.

[199] Xie Z., Zhao M., Yan C., Kong W., Lan F., Narengaowa, Zhao S., Yang Q., Bai Z., Qing H. (2023). Cathepsin B in programmed cell death machinery: Mechanisms of execution and regulatory pathways. Cell Death Dis..

[200] Chandolia R.K., Reinertsen E.M., Hansen P.J. (1999). Short communication: Lack of breed differences in responses of bovine spermatozoa to heat shock. J. Dairy Sci..

[201] Hendricks K.E., Martins L., Hansen P.J. (2009). Consequences for the bovine embryo of being derived from a spermatozoon subjected to post-ejaculatory aging and heat shock: Development to the blastocyst stage and sex ratio. J. Reprod. Dev..

[202] Sartori R., Sartor-Bergfelt R., Mertens S.A., Guenther J.N., Parrish J.J., Wiltbank M.C. (2002). Fertilization and early embryonic development in heifers and lactating cows in summer and lactating and dry cows in winter. J. Dairy Sci..

[203] Capela L., Leites I., Romão R., Lopes-da-Costa L., Pereira R. (2022). Impact of Heat Stress on Bovine Sperm Quality and Competence. Animals.

[204] Rahman M.B., Schellander K., Luceño N.L., Van Soom A. (2018). Heat stress responses in spermatozoa: Mechanisms and consequences for cattle fertility. Theriogenology.

[205] Morrell J.M. (2020). Heat stress and bull fertility. Theriogenology.

[206] Netherton J.K., Robinson B.R., Ogle R.A., Gunn A., Villaverde A.I.S.B., Colyvas K., Wise C., Russo T., Dowdell A., Baker M.A. (2022). Seasonal variation in bull semen quality demonstrates there are heat-sensitive and heat-tolerant bulls. Sci. Rep..

[207] Vanselow J., Wesenauer C., Eggert A., Sharma A., Becker F. (2024). Summer heat during spermatogenesis reduces in vitro blastocyst rates and affects sperm quality of next generation bulls. Andrology.

[208] Llamas Luceño N., de Souza Ramos Angrimani D., de Cássia Bicudo L., Szymańska K.J., Van Poucke M., Demeyere K., Meyer E., Peelman L., Mullaart E., Broekhuijse M.L.W.J. (2020). Exposing dairy bulls to high temperature-humidity index during spermatogenesis compromises subsequent embryo development in vitro. Theriogenology.

[209] Seifi-Jamadi A., Zhandi M., Kohram H., Luceño N.L., Leemans B., Henrotte E., Latour C., Demeyere K., Meyer E., Van Soom A. (2020). Influence of seasonal differences on semen quality and subsequent embryo development of Belgian Blue bulls. Theriogenology.

[210] Walters A.H., Saacke R.G., Pearson R.E., Gwazdauskas F.C. (2006). Assessment of pronuclear formation following in vitro fertilization with bovine spermatozoa obtained after thermal insulation of the testis. Theriogenology.

[211] Velazquez M.A., Idriss A., Chavatte-Palmer P., Fleming T.P. (2023). The mammalian preimplantation embryo: Its role in the environmental programming of postnatal health and performance. Anim. Reprod. Sci..

[212] Isaac E., Berg D.K., Pfeffer P.L. (2024). Using extended growth of cattle embryos in culture to gain insights into bovine developmental events on embryonic days 8 to 10. Theriogenology.

[213] Diskin M.G., Waters S.M., Parr M.H., Kenny D.A. (2016). Pregnancy losses in cattle: Potential for improvement. Reprod. Fertil. Dev..

[214] Putney D.J., Drost M., Thatcher W.W. (1988). Embryonic development in superovulated dairy cattle exposed to elevated ambient temperatures between Days 1 to 7 post insemination. Theriogenology.

[215] Vieira L.M., Rodrigues C.A., Mendanha M.F., Sá Filho M.F., Sales J.N.S., Souza A.H., Santos J.E.P., Baruselli P.S. (2014). Donor category and seasonal climate associated with embryo production and survival in multiple ovulation and embryo transfer programs in Holstein cattle. Theriogenology.

[216] Bényei B., Gáspárdy A., Cseh S. (2003). Effect of the El Nińo phenomenon on the ovarian responsiveness and embryo production of donor cows. Acta Vet. Hung..

[217] Marquez Y.C., Galina C.S., Moreno N., Ruiz H., Ruiz A., Merchant H. (2005). Seasonal effect on zebu embryo quality as determined by their degree of apoptosis and resistance to cryopreservation. Reprod. Domest. Anim..

[218] Martínez J.F., Galina C.S., Ortiz P., Maquivar M.G., Romero-Zúñiga J.J. (2021). Effects of Season on Donor and Recipient Cows and Calf Performance from Birth to Weaning in Embryo Transfer Programs in the Tropics. Animals.

[219] Kamano S., Ikeda S., Sugimoto M., Kume S. (2014). The effects of calcitonin on the development of and Ca^2+^ levels in heat-shocked bovine preimplantation embryos in vitro. J. Reprod. Dev..

[220] Ortega M.S., Rocha-Frigoni N.A.S., Mingoti G.Z., Roth Z., Hansen P.J. (2016). Modification of embryonic resistance to heat shock in cattle by melatonin and genetic variation in HSPA1L. J. Dairy Sci..

[221] Rivera R.M., Kelley K.L., Erdos G.W., Hansen P.J. (2003). Alterations in ultrastructural morphology of two-cell bovine embryos produced in vitro and in vivo following a physiologically relevant heat shock. Biol. Reprod..

[222] Block J., Chase C.C., Hansen P.J. (2002). Inheritance of resistance of bovine preimplantation embryos to heat shock: Relative importance of the maternal versus paternal contribution. Mol. Reprod. Dev..

[223] Paula-Lopes F.F., Chase C.C., Al-Katanani Y.M., Krininger C.E., Rivera R.M., Tekin S., Majewski A.C., Ocon O.M., Olson T.A., Hansen P.J. (2003). Genetic divergence in cellular resistance to heat shock in cattle: Differences between breeds developed in temperate versus hot climates in responses of preimplantation embryos, reproductive tract tissues and lymphocytes to increased culture temperatures. Reproduction.

[224] Sakatani M., Kobayashi S., Takahashi M. (2004). Effects of heat shock on in vitro development and intracellular oxidative state of bovine preimplantation embryos. Mol. Reprod. Dev..

[225] Paula-Lopes F.F., Hansen P.J. (2002). Apoptosis is an adaptive response in bovine preimplantation embryos that facilitates survival after heat shock. Biochem. Biophys. Res. Commun..

[226] Sugiyama S., McGowan M., Kafi M., Phillips N., Young M. (2003). Effects of increased ambient temperature on the development of in vitro derived bovine zygotes. Theriogenology.

[227] Hernández-Cerón J., Chase C.C., Hansen P.J. (2004). Differences in Heat Tolerance Between Preimplantation Embryos from Brahman, Romosinuano, and Angus Breeds. J. Dairy Sci..

[228] Jousan F.D., Hansen P.J. (2004). Insulin-like growth factor-I as a survival factor for the bovine preimplantation embryo exposed to heat shock. Biol. Reprod..

[229] Rivera R.M., Dahlgren G.M., De Castro E.P.L.A., Kennedy R.T., Hansen P.J. (2004). Actions of thermal stress in two-cell bovine embryos: Oxygen metabolism, glutathione and ATP content, and the time-course of development. Reproduction.

[230] Jousan F.D., Hansen P.J. (2007). Insulin-like growth factor-I promotes resistance of bovine preimplantation embryos to heat shock through actions independent of its anti-apoptotic actions requiring PI3K signaling. Mol. Reprod. Dev..

[231] Sakatani M., Suda I., Oki T., Kobayashi S., Kobayashi S., Takahashi M. (2007). Effects of purple sweet potato anthocyanins on development and intracellular redox status of bovine preimplantation embryos exposed to heat shock. J. Reprod. Dev..

[232] de Castro e Paula L.A., Hansen P.J. (2008). Modification of actions of heat shock on development and apoptosis of cultured preimplantation bovine embryos by oxygen concentration and dithiothreitol. Mol. Reprod. Dev..

[233] Namekawa T., Ikeda S., Sugimoto M., Kume S. (2010). Effects of astaxanthin-containing oil on development and stress-related gene expression of bovine embryos exposed to heat stress. Reprod. Domest. Anim..

[234] Bonilla A.Q., Oliveira L.J., Ozawa M., Newsom E.M., Lucy M.C., Hansen P.J. (2011). Developmental changes in thermoprotective actions of insulin-like growth factor-1 on the preimplantation bovine embryo. Mol. Cell Endocrinol..

[235] Kuroki T., Ikeda S., Okada T., Maoka T., Kitamura A., Sugimoto M., Kume S. (2013). Astaxanthin ameliorates heat stress-induced impairment of blastocyst development in vitro:--astaxanthin colocalization with and action on mitochondria. J. Assist. Reprod. Genet..

[236] Silva C.F., Sartorelli E.S., Castilho A.C.S., Satrapa R.A., Puelker R.Z., Razza E.M., Ticianelli J.S., Eduardo H.P., Loureiro B., Barros C.M. (2013). Effects of heat stress on development, quality and survival of Bos indicus and Bos taurus embryos produced in vitro. Theriogenology.

[237] Khatun H., Egashira J., Sakatani M., Takenouchi N., Tatemoto H., Wada Y., Yamanaka K.I. (2018). Sericin enhances the developmental competence of heat-stressed bovine embryos. Mol. Reprod. Dev..

[238] Stamperna K., Giannoulis T., Dovolou E., Kalemkeridou M., Nanas I., Dadouli K., Moutou K., Mamuris Z., Amiridis G.S. (2021). The Effects of Heat Shock Protein 70 Addition in the Culture Medium on the Development and Quality of In Vitro Produced Heat Shocked Bovine Embryos. Animals.

[239] Edwards J.L., King W.A., Kawarsky S.J., Ealy A.D. (2001). Responsiveness of early embryos to environmental insults: Potential protective roles of HSP70 and glutathione. Theriogenology.

[240] Edwards J.L., Ealy A.D., Monterroso V.H., Hansen P.J. (1997). Ontogeny of temperature-regulated heat shock protein 70 synthesis in preimplantation bovine embryos. Mol. Reprod. Dev..

[241] Chandolia R.K., Peltier M.R., Tian W., Hansen P.J. (1999). Transcriptional control of development, protein synthesis, and heat-induced heat shock protein 70 synthesis in 2-cell bovine embryos. Biol. Reprod..

[242] Al-Katanani Y.M., Hansen P.J. (2002). Induced thermotolerance in bovine two-cell embryos and the role of heat shock protein 70 in embryonic development. Mol. Reprod. Dev..

[243] Oliveira C.S., Marques S.C.S., Guedes P.H.E., Feuchard V.L., Camargo A.J.R., de Freitas C., Camargo L.S.A. (2021). Thermal-treatment protocol to induce thermotolerance in bovine embryos. Reprod. Fertil. Dev..

[244] Jousan F.D., Oliveira L.J., Hansen P.J. (2008). Short-Term culture of in vitro produced bovine preimplantation embryos with insulin-like growth factor-i prevents heat shock-induced apoptosis through activation of the Phosphatidylinositol 3-Kinase/Akt pathway. Mol. Reprod. Dev..

[245] Paula-Lopes F.F., Hansen P.J. (2002). Heat shock-induced apoptosis in preimplantation bovine embryos is a developmentally regulated phenomenon. Biol. Reprod..

[246] Malayer J.R., Hansen P.J., Buhi W.C. (1988). Effect of day of the oestrous cycle, side of the reproductive tract and heat shock on in-vitro protein secretion by bovine endometrium. J. Reprod. Fertil..

[247] Malayer J.R., Hansen P.J. (1990). Differences between Brahman and Holstein cows in heat-shock induced alterations of protein synthesis and secretion by oviducts and uterine endometrium1. J. Anim. Sci..

[248] Putney D.J., Malayer J.R., Gross T.S., Thatcher W.W., Hansen P.J., Drost M. (1988). Heat Stress-Induced Alterations in the Synthesis and Secretion of Proteins and Prostaglandins by Cultured Bovine Conceptuses and Uterine Endometrium1. Biol. Reprod..

[249] Sakai S., Hagihara N., Kuse M., Kimura K., Okuda K. (2018). Heat stress affects prostaglandin synthesis in bovine endometrial cells. J. Reprod. Dev..

[250] Sakai S., Yagi M., Fujime N., Kuse M., Sakumoto R., Yamamoto Y., Okuda K., Kimura K. (2021). Heat stress influences the attenuation of prostaglandin synthesis by interferon tau in bovine endometrial cells. Theriogenology.

[251] Chotimanukul S., Suwimonteerabutr J., Techakumphu M., Swangchan-Uthai T. (2022). In Vitro Effects of Short-Term and Long-Term Heat Exposures on the Immune Response and Prostaglandin Biosynthesis in Bovine Endometrial Cells. Animals.

[252] Sakai S., Hatabu T., Yamamoto Y., Kimura K. (2020). Alteration of chemokine production in bovine endometrial epithelial and stromal cells under heat stress conditions. Physiol. Rep..

[253] Sakai S., Inoue Y., Tanaka K., Yamamoto Y., Iwata H., Kimura K. (2022). Hyperthermia alters interleukin-6 production in response to lipopolysaccharide via endoplasmic reticulum stress in bovine endometrial cells. J. Cell. Physiol..

[254] Molinari P.C.C., Bromfield J.J. (2023). Inflammatory responses of bovine endometrial epithelial cells are increased under in vitro heat stress conditions. J. Therm. Biol..

[255] Murata H., Kunii H., Kusama K., Sakurai T., Bai H., Kawahara M., Takahashi M. (2021). Heat stress induces oxidative stress and activates the KEAP1-NFE2L2-ARE pathway in bovine endometrial epithelial cells. Biol. Reprod..

[256] Sponchiado M., Gonella-Diaza A.M., Rocha C.C., Turco E.G.L., Pugliesi G., Leroy J.L.M.R., Binelli M. (2019). The pre-hatching bovine embryo transforms the uterine luminal metabolite composition in vivo. Sci. Rep..

[257] Husnain A., Arshad U., Zimpel R., Schmitt E., Dickson M.J., Perdomo M.C., Marinho M.N., Ashrafi N., Graham S.F., Bishop J.V. (2023). Induced endometrial inflammation compromises conceptus development in dairy cattle. Biol. Reprod..

[258] Roman-Ponce H., Thatcher W.W., Caton D., Barron D.H., Wilcox C.J. (1978). Thermal stress effects on uterine blood flow in dairy cows. J. Anim. Sci..

[259] Katagiri S., Moriyoshi M. (2013). Alteration of the endometrial EGF profile as a potential mechanism connecting the alterations in the ovarian steroid hormone profile to embryonic loss in repeat breeders and high-producing cows. J. Reprod. Dev..

[260] Kawano K., Yanagawa Y., Nagano M., Katagiri S. (2022). Effects of heat stress on the endometrial epidermal growth factor profile and fertility in dairy cows. J. Reprod. Dev..

[261] Nishisozu T., Singh J., Abe A., Okamura K., Dochi O. (2023). Effects of the temperature-humidity index on conception rates in Holstein heifers and cows receiving in vitro-produced Japanese Black cattle embryos. J. Reprod. Dev..

[262] Abdel Aziz R.L., Hussein M.M., Mohamed M.A.A., Elsaid H., Abdel-Wahab A. (2022). Heat stress during critical windows of the oestrous cycle and risk of pregnancy establishment in embryo-recipient dairy heifers. Reprod. Domest. Anim..

[263] Maillo V., Rizos D., Besenfelder U., Havlicek V., Kelly A.K., Garrett M., Lonergan P. (2012). Influence of lactation on metabolic characteristics and embryo development in postpartum Holstein dairy cows. J. Dairy Sci..

[264] Assel A., Besenfelder U., Wagener K., Allram J., Tekin M., Vogl C., Drillich M., Havlicek V. (2024). Is the proteome of the oviductal fluid in dairy cows affected by heat stress?. Reprod. Fertil. Dev..

[265] Islam M.A., Lomax S., Doughty A.K., Islam M.R., Thomson P.C., Clark C.E.F. (2023). Revealing the diversity of internal body temperature and panting response for feedlot cattle under environmental thermal stress. Sci. Rep..

[266] Nanas I., Chouzouris T.M., Dadouli K., Dovolou E., Stamperna K., Barbagianni M., Valasi I., Tsiaras A., Amiridis G.S. (2020). A study on stress response and fertility parameters in phenotypically thermotolerant and thermosensitive dairy cows during summer heat stress. Reprod. Domest. Anim..

[267] Chen X., Shu H., Sun F., Yao J., Gu X. (2023). Impact of Heat Stress on Blood, Production, and Physiological Indicators in Heat-Tolerant and Heat-Sensitive Dairy Cows. Animals.

[268] Hansen P.J. (2004). Physiological and cellular adaptations of zebu cattle to thermal stress. Anim. Reprod. Sci..

[269] Hansen P.J. (2014). Genetic variation in resistance of the preimplantation bovine embryo to heat shock. Reprod. Fertil. Dev..

[270] Barros C.M., Pegorer M.F., Vasconcelos J.L.M., Eberhardt B.G., Monteiro F.M. (2006). Importance of sperm genotype (indicus versus taurus) for fertility and embryonic development at elevated temperatures. Theriogenology.

[271] Paula-Lopes F.F., Lima R.S., Satrapa R.A., Barros C.M. (2013). Physiology and Endocrinology Symposium: Influence of cattle genotype (Bos indicus vs. Bos taurus) on oocyte and preimplantation embryo resistance to increased temperature. J. Anim. Sci..

[272] Satrapa R.A., Nabhan T., Silva C.F., Simões R.A.L., Razza E.M., Puelker R.Z., Trinca L.A., Barros C.M. (2011). Influence of sire breed (Bos indicus versus Bos taurus) and interval from slaughter to oocyte aspiration on heat stress tolerance of in vitro-produced bovine embryos. Theriogenology.

[273] Camargo L.S.A., Viana J.H.M., Ramos A.A., Serapião R.V., de Sa W.F., Ferreira A.M., Guimarães M.F.M., do Vale Filho V.R. (2007). Developmental competence and expression of the Hsp 70.1 gene in oocytes obtained from Bos indicus and Bos taurus dairy cows in a tropical environment. Theriogenology.

[274] Ticianelli J.S., Emanuelli I.P., Satrapa R.A., Castilho A.C.S., Loureiro B., Sudano M.J., Fontes P.K., Pinto R.F.P., Razza E.M., Surjus R.S. (2017). Gene expression profile in heat-shocked Holstein and Nelore oocytes and cumulus cells. Reprod. Fertil. Dev..

[275] Eberhardt B.G., Satrapa R.A., Capinzaiki C.R., Trinca L.A., Barros C.M. (2009). Influence of the breed of bull (Bos taurus indicus vs. Bos taurus taurus) and the breed of cow (Bos taurus indicus, Bos taurus taurus and crossbred) on the resistance of bovine embryos to heat. Anim. Reprod. Sci..

[276] Shen P.C., Lee J.W., Cheng W.T.K., Su H.Y., Lee S.N., Liu B.T., Wang C.H., Chen L.R., Ju J.C. (2010). Differential thermal sensitivity between the recipient ooplasm and the donor nucleus in Holstein and Taiwan native yellow cattle. Theriogenology.

[277] Lee J.-W., Li H., Wu H.-Y., Liu S.-S., Shen P.-C. (2016). Improved cellular thermotolerance in cloned Holstein cattle derived with cytoplasts from a thermotolerant breed. Theriogenology.

[278] Lee Y.-J., Lin W., Peng S.-Y., Lee J.-W., Lin Y.-H., Yu C., Shen P.-C. (2022). Effects of intracytoplasmic sperm injection timing and fertilization methods on the development of bovine spindle transferred embryos. Theriogenology.

[279] Lee Y.-J., Lee J.-W., Huang C.-W., Yang K.-T., Peng S.-Y., Yu C., Lee Y.-H., Lai I.-L., Shen P.-C. (2024). Identification of Molecular Profile of Ear Fibroblasts Derived from Spindle-Transferred Holstein Cattle with Ooplasts from Taiwan Yellow Cattle under Heat Stress. Animals.

[280] Toledo I.M., Fabris T.F., Tao S., Dahl G.E. (2020). When do dry cows get heat stressed? Correlations of rectal temperature, respiration rate, and performance. JDS Commun..

[281] Correa-Calderon A., Armstrong D., Ray D., DeNise S., Enns M., Howison C. (2004). Thermoregulatory responses of Holstein and Brown Swiss heat-stressed dairy cows to two different cooling systems. Int. J. Biometeorol..

[282] Dalcin V.C., Fischer V., Daltro D.d.S., Alfonzo E.P.M., Stumpf M.T., Kolling G.J., Silva M.V.G.B.d., McManus C. (2016). Physiological parameters for thermal stress in dairy cattle. Rev. Bras. De Zootec..

[283] da Costa A.N.L., Feitosa J.V., Montezuma P.A., de Souza P.T., de Araújo A.A. (2015). Rectal temperatures, respiratory rates, production, and reproduction performances of crossbred Girolando cows under heat stress in northeastern Brazil. Int. J. Biometeorol..

[284] Silva Leles J., Campos Salles Rodrigues I., Vieira Neto M.F., Martins Viana Neto A., Ramos da Rocha D., Lima da Costa A.N., Gorete Flores Salles M., Alencar de Araújo A. (2017). Heat Stress and Body Temperature in Brown Swiss Cows Raised in Semi-Arid Climate of Ceará State, Brazil. Acta Sci. Vet..

[285] Oliveira A.V.D., Reis E.M.B., Ferraz P.F.P., Barbari M., Santos G.S., Cruz M.V.R., Silva G.F., Silva A.O.L. (2022). Effects of thermal environment on dairy cattle under a grazing system in the Western Amazon, Brazil. Arq. Bras. De Med. Veterinária E Zootec..

[286] Mohamed Jafar A.-H. (2018). The Effects of Evaporative Cooling on Heat Stressed Dairy Holstein Cows Under a Semi-Arid Environment in Riyadh Area, Saudi Arabia. Anim. Vet. Sci..

[287] Djelailia H., M’Hamdi N., Bouraoui R., Najar T. (2021). Effects of thermal stress on physiological state and hormone concentrations in Holstein cows under arid climatic conditions. S. Afr. J. Anim. Sci..

[288] Corazzin M., Saccà E., Lippe G., Romanzin A., Foletto V., Da Borso F., Piasentier E. (2020). Effect of Heat Stress on Dairy Cow Performance and on Expression of Protein Metabolism Genes in Mammary Cells. Animals.

[289] Cartwright S., Schmied J., Livernois A., Mallard B.A. (2022). Physiological Response to Heat Stress in Immune Phenotyped Canadian Holstein Dairy Cattle in Free-Stall and Tie-Stall Management Systems. Front. Anim. Sci..

[290] Yan G., Liu K., Hao Z., Shi Z., Li H. (2021). The effects of cow-related factors on rectal temperature, respiration rate, and temperature-humidity index thresholds for lactating cows exposed to heat stress. J. Therm. Biol..

[291] Luo H., Brito L.F., Li X., Su G., Dou J., Xu W., Yan X., Zhang H., Guo G., Liu L. (2021). Genetic parameters for rectal temperature, respiration rate, and drooling score in Holstein cattle and their relationships with various fertility, production, body conformation, and health traits. J. Dairy Sci..

[292] Abbott C.R., Saxton A.M., Rispoli L.A., Payton R.R., Pohler K.G., Schrick F.N., Edwards J.L. (2018). An in vivo model to assess the thermoregulatory response of lactating Holsteins to an acute heat stress event occurring after a pharmacologically-induced LH surge. J. Therm. Biol..

[293] Gwazdauskas F.C. (1985). Effects of climate on reproduction in cattle. J. Dairy Sci..

[294] Wrenn T.R., Bitman J., Sykes J.F. (1958). Body Temperature Variations in Dairy Cattle during the Estrous Cycle and Pregnancy. J. Dairy Sci..

[295] Kendall P.E., Webster J.R. (2009). Season and physiological status affects the circadian body temperature rhythm of dairy cows. Livest. Sci..

[296] Suthar V.S., Burfeind O., Patel J.S., Dhami A.J., Heuwieser W. (2011). Body temperature around induced estrus in dairy cows. J. Dairy Sci..

[297] Wang S., Zhang H., Tian H., Chen X., Li S., Lu Y., Li L., Wang D. (2020). Alterations in vaginal temperature during the estrous cycle in dairy cows detected by a new intravaginal device—A pilot study. Trop. Anim. Health Prod..

[298] Sakatani M., Balboula A.Z., Yamanaka K., Takahashi M. (2012). Effect of summer heat environment on body temperature, estrous cycles and blood antioxidant levels in Japanese Black cow. Anim. Sci. J..

[299] Mills M.D., Pollock A.B., Batey I.E., O’Neil M.A., Schrick F.N., Payton R.R., Moorey S.E., Fioravanti P., Hipsher W., Zoca S.M. (2024). Magnitude and persistence of higher estrus-associated temperatures in beef heifers and suckled cows. J. Anim. Sci..

[300] Vickers L.A., Burfeind O., von Keyserlingk M.A., Veira D.M., Weary D.M., Heuwieser W. (2010). Technical note: Comparison of rectal and vaginal temperatures in lactating dairy cows. J. Dairy Sci..

[301] Burfeind O., Suthar V.S., Heuwieser W. (2012). Effect of heat stress on body temperature in healthy early postpartum dairy cows. Theriogenology.

[302] Dikmen S., Mateescu R.G., Elzo M.A., Hansen P.J. (2018). Determination of the optimum contribution of Brahman genetics in an Angus-Brahman multibreed herd for regulation of body temperature during hot weather. J. Anim. Sci..

[303] Sarlo Davila K.M., Hamblen H., Hansen P.J., Dikmen S., Oltenacu P.A., Mateescu R.G. (2019). Genetic parameters for hair characteristics and core body temperature in a multibreed Brahman-Angus herd1. J. Anim. Sci..

[304] Carvalheira L.R., Wenceslau R.R., Ribeiro L.D.S., de Carvalho B.C., Borges Á.M., Camargo L.S.A. (2021). Daily vaginal temperature in Girolando cows from three different genetic composition under natural heat stress. Transl. Anim. Sci..

[305] Pontiggia A., Münger A., Eggerschwiler L., Holinger M., Stucki D., Ammer S., Bruckmaier R.M., Dohme-Meier F., Keil N.M. (2024). Behavioural responses related to increasing core body temperature of grazing dairy cows experiencing moderate heat stress. Animal.

[306] Rashamol V., Sejian V., Bagath M., Krishnan G., Archana P., Bhatta R. (2018). Physiological adaptability of livestock to heat stress: An updated review. J. Anim. Behav. Biometeorol..

[307] Sejian V., Bhatta R., Gaughan J.B., Dunshea F.R., Lacetera N. (2018). Review: Adaptation of animals to heat stress. Animal.

[308] Brown-Brandl T.M. (2018). Understanding heat stress in beef cattle. Rev. Bras. De Zootec..

[309] Vitali A., Grossi G., Lacetera N. (2024). Vaginal temperature of lactating cows during heat waves or normal summer day and effect of additional daily cooling treatments as heat load mitigation strategy. Int. J. Biometeorol..

[310] Vermunt J., Tranter B. Heat stress in dairy cattle—A review, and some of the potential risks associated with the nutritional management of this condition. Proceedings of the Annual Conference of the Australian Veterinary Association—Queensland Division.

[311] Hunter R.H.F., López-Gatius F. (2020). Temperature gradients in the mammalian ovary and genital tract: A clinical perspective. Eur. J. Obstet. Gynecol. Reprod. Biol..

[312] López-Gatius F., Hunter R.H.F. (2020). Local cooling of the ovary and its implications for heat stress effects on reproduction. Theriogenology.

[313] López-Gatius F., Hunter R.H.F. (2019). Pre-ovulatory follicular cooling correlates positively with the potential for pregnancy in dairy cows: Implications for human IVF. J. Gynecol. Obstet. Hum. Reprod..

[314] López-Gatius F., Hunter R.H.F. (2019). Pre-ovulatory follicular temperature in bi-ovular cows. J. Reprod. Dev..

[315] López-Gatius F., Hunter R. (2017). Clinical relevance of pre-ovulatory follicular temperature in heat-stressed lactating dairy cows. Reprod. Domest. Anim..

[316] Fallon G.R. (1962). Body Temperature and Fertilization in the Cow. Reproduction.

[317] Liles H.L., Schneider L.G., Pohler K.G., Oliveira Filho R.V., Neal Schrick F., Payton R.R., Rhinehart J.D., Thompson K.W., McLean K., Edwards J.L. (2022). Positive relationship of rectal temperature at fixed timed artificial insemination on pregnancy outcomes in beef cattle. J. Anim. Sci..

[318] Pollock A.B., Moorey S.E., Hessock E.A., Klabnik J.L., Payton R.R., Schrick F.N., Campagna S.R., Edwards J.L. (2023). Relationship between higher estrus-associated temperatures and the bovine preovulatory follicular fluid metabolome. Front. Anim. Sci..

[319] López-Gatius F., Garcia-Ispierto I., Hunter R.H.F. (2021). Cervix-rectum temperature differential at the time of insemination is correlated with the potential for pregnancy in dairy cows. J. Reprod. Dev..

[320] Matoba S., Yoshioka H., Matsuda H., Sugimura S., Aikawa Y., Ohtake M., Hashiyada Y., Seta T., Nakagawa K., Lonergan P. (2014). Optimizing production of in vivo-matured oocytes from superstimulated Holstein cows for in vitro production of embryos using X-sorted sperm. J. Dairy Sci..

[321] Grinsted J., Kjer J.J., Blendstrup K., Pedersen J.F. (1985). Is low temperature of the follicular fluid prior to ovulation necessary for normal oocyte development?. Fertil. Steril..

[322] Sherbahn R. (2010). Assessment of effect of follicular fluid temperature at egg retrieval on blastocyst development, implantation and live birth rates. Fertil. Steril..

[323] Da Broi M.G., Giorgi V.S.I., Wang F., Keefe D.L., Albertini D., Navarro P.A. (2018). Influence of follicular fluid and cumulus cells on oocyte quality: Clinical implications. J. Assist. Reprod. Genet..

[324] Piccione G., Caola G., Refinetti R. (2003). Daily and estrous rhythmicity of body temperature in domestic cattle. BMC Physiol..

[325] El-Sheikh Ali H., Kitahara G., Tamura Y., Kobayashi I., Hemmi K., Torisu S., Sameshima H., Horii Y., Zaabel S., Kamimura S. (2013). Presence of a temperature gradient among genital tract portions and the thermal changes within these portions over the estrous cycle in beef cows. J. Reprod. Dev..

[326] Gwazdauskas F.C., Thatcher W.W., Wilcox C.J. (1973). Physiological, environmental, and hormonal factors at insemination which may affect conception. J. Dairy Sci..

[327] El-Sheikh Ali H., Tamura Y., Sameshima H., Kitahara G. (2020). Impact of summer heat stress on the thermal environment of bovine female genital tract. Trop. Anim. Health Prod..

[328] García-Martínez S., Latorre R., Sánchez-Hurtado M.A., Sánchez-Margallo F.M., Bernabò N., Romar R., López-Albors O., Coy P. (2020). Mimicking the temperature gradient between the sow’s oviduct and uterus improves in vitro embryo culture output. Mol. Hum. Reprod..

[329] Hino T., Yanagimachi R. (2019). Active peristaltic movements and fluid production of the mouse oviduct: Their roles in fluid and sperm transport and fertilization. Biol. Reprod..

[330] Besenfelder U., Brem G., Havlicek V. (2020). Review: Environmental impact on early embryonic development in the bovine species. Animal.

[331] Roth Z. (2020). Reproductive physiology and endocrinology responses of cows exposed to environmental heat stress—Experiences from the past and lessons for the present. Theriogenology.

[332] Moura M.T., Paula-Lopes F.F. (2020). Thermoprotective molecules to improve oocyte competence under elevated temperature. Theriogenology.

[333] Abdelnour S.A., Yang C.-Y., Swelum A.A., Abd El-Hack M.E., Khafaga A.F., Abdo M., Shang J.-H., Lu Y.-Q. (2020). Molecular, functional, and cellular alterations of oocytes and cumulus cells induced by heat stress and shock in animals. Environ. Sci. Pollut. Res..

[334] Swain J.E. (2012). Is there an optimal pH for culture media used in clinical IVF?. Hum. Reprod. Update.

[335] Swain J.E. (2010). Optimizing the culture environment in the IVF laboratory: Impact of pH and buffer capacity on gamete and embryo quality. Reprod. Biomed. Online.

[336] FitzHarris G., Baltz J.M. (2009). Regulation of intracellular pH during oocyte growth and maturation in mammals. Reproduction.

[337] Wale P.L., Gardner D.K. (2015). The effects of chemical and physical factors on mammalian embryo culture and their importance for the practice of assisted human reproduction. Hum. Reprod. Update.

[338] Gatimel N., Moreau J., Parinaud J., Léandri R.D. (2020). Need for choosing the ideal pH value for IVF culture media. J. Assist. Reprod. Genet..

